# Supplementary material for: Prevalence of yaws and syphilis in the Ashanti region of Ghana and occurrence of H. ducreyi, herpes simplex virus 1 and herpes simplex virus 2 in skin lesions associated with treponematoses
Source: PLoS One. 2024 May 22;19(5):e0295088. doi: 10.1371/journal.pone.0295088 (PMC11111032; doi:10.1371/journal.pone.0295088)
Supplement: S3 File — (PDF) [file pone.0295088.s006.pdf]

Boaitey et al. PONE-D-23-37271R1

Supporting Information - S3 File

In this study, we reported the seroprevalence of yaws and syphilis, caused by *Treponema pallidum pertenue* and *Treponema pallidum pallidum*, respectively, using a point-of-care test in 156 participants recruited in the Ashanti region of Ghana, one of the 15 countries where yaws is endemic. Alongside the seroprevalence of *T. pallidum* in the lesions sampled, we identified the presence of three pathogens, *H. ducreyi*, herpes simplex virus 1 (HSV-1) and 2 (HSV-2), known to occur in skin lesions that can be indistinguishable from those caused by *T. pallidum*. This was achieved using a multiplex-PCR approach, originally designed to identify these pathogens from genital lesions [1].

In this supporting information file, we discuss our findings in relation to (i) other studies that have independently reported the prevalence of yaws and syphilis in different countries; (ii) the variety of communities affected by skin ulcers within a single region; (iii) congenital syphilis; (iv) recombination of distinct *Treponema* species and subspecies and how this can affect diagnosis and treatment. All of these aspects affect policies focused on the eradication of yaws and the elimination of syphilis and therefore require further consideration.

Material and methods

The prevalence of yaws and syphilis collected from previously published research

Information from peer-reviewed articles reporting the prevalence of yaws and syphilis was collected using the following search engines: University of Westminster Library Search, Science Direct, PubMed, MEDLINE, Google Scholar and Web of Science ; for the following set of words and expressions (“yaws” OR “treponematosis” AND “prevalence OR “incidence” OR “diagnosis”) OR

(“yaws” AND [each current yaws-endemic country]) and (“syphilis” OR “treponematosis” AND “prevalence” OR “incidence” OR “diagnoses”). Studies on the prevalence of yaws and syphilis can vary greatly in the way they have been conducted. Therefore, we specifically selected articles that provided information on (i) the country where the study was conducted, (ii) the number of positive cases and the studied sample size. These were used to infer the prevalence of the corresponding treponematose per country. For each study and each country, the prevalence of yaws and syphilis was calculated as the ratio between the number of participants whose serological test was positive over the total number of participants screened. For example, even though the study of Boock et al. [2] showed the number of positive cases for yaws, we chose not to include this study into the set of peer-reviewed articles selected, because the sample size of people screened was not explicitly given. This ratio calculation makes this inferred prevalence comparable to the one observed in the Ashanti region. We further recorded (iii) the diagnostic method(s) used (either clinical or at least one serological test), (iv) dates when studies were conducted, started and/or ended (if different), and (v) specifically for syphilis, whether specific identifiable groups were investigated (i.e. blood donors (BD), men who have sex with men (MSM), female sex workers (FSW), drug users (DU) and inmates (prisoners)). Articles that fit these criteria were included, regardless of whether they had been conducted in yaws endemic countries or not (S2 Table). This enabled sampling information on the prevalence of both treponematoses that was not geographically restricted and allowed a fairer comparison between the two.

Statistical analyses and data visualisations

Statistical analyses were performed with R (version 4.2.2) on R Studio version 2022.07.1 Build 554. Figures were produced with the ggplot2 package (version 3.4.0) and maps were obtained using additional R packages, including rworldmap, maps, raster, countrycode, viridis, scales, dplyr, cowplot. R scripts used to produce the Figures presented in the manuscript and the Supplementary information files are available as Supporting Information (S2 File).

Results

Comparison of the prevalence of yaws and syphilis observed in this study and from previously published research

S1 Fig shows the prevalence of yaws and syphilis estimated from previously published studies and how they compare to those observed in this study. A total of 89 peer-reviewed research articles were included in this comparison. Twenty-eight (28) articles related to the prevalence of yaws and 61 articles related to the prevalence of syphilis (see S2 Table), covering a total of 39 countries. Significantly more studies on the prevalence of syphilis have been published than on the prevalence of yaws between 1991 and 2020 (Chi-square test p-value = 0.0005).

Noteworthy, there is no significant difference between any of the estimated prevalence of yaws and syphilis (S1A Fig). The Wilcoxon Rank Sum test p-values are 0.77 between the published prevalence of yaws and syphilis (globally); 0.67 between the published prevalence of yaws and syphilis in Ecuador; 0.70 between the published prevalence of yaws and syphilis in Ghana; 1 between the published prevalence of yaws and syphilis in Indonesia; 0.50 between the published prevalence of yaws and syphilis in Nigeria. The Chi-squared test p-value is 0.44 between the observed seroprevalence of yaws and syphilis in the Ashanti region (this study, S1 Table). Out of the 15 countries known to be endemic for yaws, 13 are represented in the selected peer-reviewed articles reporting the prevalence of yaws or syphilis (no data published between 1991 and 2020 on the prevalence of yaws and syphilis from Cameroon and Liberia that fitted our criteria). Only four countries showed estimates of the prevalence of yaws and syphilis, but from independent studies, i.e. Ecuador, Ghana, Indonesia, and Nigeria (S1B and S1C Fig).

Discussion

The prevalence of yaws and syphilis in yaws-endemic countries

We show the seroprevalence of yaws and the seroprevalence of syphilis in the Ashanti region do not differ significantly. We compared this finding to other published studies that have reported the seroprevalence of yaws and syphilis in various countries (S1B and S1C Fig). This showed our finding falls into a more general trend, where, whether the comparison is global (worldwide), or local (in yaws-endemic countries), the seroprevalence of these treponematoses do not differ (S1A Fig). The collection of articles (S2 Table) to make this comparison however highlighted that, to our knowledge, our study (Table 2) is the first to report the prevalence of yaws and syphilis together, from a single region.

Various communities affected by skin ulcers

The group that was disproportionately affected by yaws in this geographical region were the Northerners (S1 Table). The Northerners in this area are people from the Upper East, Upper West and Northern regions of Ghana (Fig 1B). Most people from these regions migrate and settle in rural communities in the Ashanti region, in search of jobs, where their living environment may still be precarious, consequently promoting their exposure to several infectious diseases [3]. This may explain why most of the participants with skin lesions consistent with yaws were sampled from these deprived communities of the Ashanti region, since yaws is known to be associated with poverty and poor hygiene [4,5]. This highlights that within a single region, and even within study sites, the distribution of yaws is not homogeneous. Distinct communities present skin ulcers, which may be caused by the same or different pathogens. Understanding the communities specifically affected by pathogens diagnosed with sensitive approaches will improve management and control of the pathogens causing these ulcers.

T. pallidum infections and congenital syphilis

The 10.8% *T. pallidum* seroprevalence among study participants with syphilis-like lesions confirms active or latent syphilis in Ghana (Table 1). In this study, the median age of participants with syphilis-like lesions was 29 with minimum age of 19 and maximum age of 75, a sexually active age

range. These infected participants could transmit their *T. pallidum* infection to an unborn baby, leading to congenital syphilis. WHO considers congenital syphilis as a public health problem [6]. To eliminate congenital syphilis, treating pregnant women is not sufficient: syphilis must also be eliminated from the general population. In fact, reducing the global syphilis incidence by 90% between 2018 and 2030 is one of the four ambitious targets detailed in the Global Health Sector strategy addressing sexually transmitted diseases [7]. We argue supporting accurate diagnosis of syphilis and specific assessment of the presence of its causative treponeme, *T. pallidum pallidum*, and of related treponemes, such as *T. pallidum pertenue* causing yaws, in countries such as Ghana, will be essential to achieving this Global Health Sector strategy target.

The importance of recombination in diagnosis and treatment

In this study, we mentioned one of the participants whose yaws-like lesion was positive for *T. pallidum* with the multiplex PCR, suggesting an early stage of the infection. This participant was 17 years old, an age when an individual could also be sexually active, and therefore can potentially be infected with syphilis treponemes. Even though this is speculative, this may be an opportunity of co-infection and circulation of two treponeme species, i.e. *T. pallidum pertenue* and *T. pallidum pallidum*, within one host. Such a situation would enable recombination of yaws and syphilis treponemes.

While numerous studies [8-12] have evidenced intergenomic recombination within *T. pallidum* subspecies as well as between species, cases of co-infections have not been explicitly suggested or observed. These cases however pose diagnostic challenges because variants resulting from recombination may lead to changes in conserved regions of DNA/RNA or proteins [8-12], which tend to be the molecular targets of both serological and molecular diagnoses [11,13]. In those situations, antigens (used in serological tests) may not react properly with antibodies produced by an individual infected by a strain resulting from the recombination of two *T. pallidum* subspecies, inducing a false negative diagnosis [11,13]. Indeed, Marks and colleagues [13] reported that Solomons island strains of *T. pallidum pertenue* were undetectable by the Centers for Disease Control and Prevention

(CDC) diagnostic PCR assay (2015 CDC real-time PCR assay) used in several studies. They reported this was due to recombinant regions in Solomon Island strains, where single nucleotide polymorphisms in the tp0858 gene region introduced sequence variation in the primer binding site of this CDC assay.

It is worth mentioning here that the approaches we have applied do not allow distinguishing the *T. pallidum* subspecies that could have caused the lesions. We can consequently not exclude that the observed cases of yaws-like lesions (caused by *T. pallidum pertenue*) might actually be cases of bejel (caused by *T. pallidum endemicum*). We can only suggest that the presence of *T. pallidum endemicum* is unlikely here, because it has not been previously reported in Ghana (see the main text Discussion and [14-16]).

Conclusion

Our study is the first to report the seroprevalence of both treponematoses yaws and syphilis, in a single region, the Ashanti region of Ghana (S1A Fig, S1 and S2 Tables). While syphilis and yaws affect different cohorts (sexually active adults for syphilis and children for yaws), they are caused by bacteria that are 99.8% genetically similar [17]. This, together with evidences of recombination between strains within *Treponema* subspecies and between species, argues for both treponematoses to be diagnosed accurately and treated appropriately in regions where yaws is endemic, such as Ghana.

In addition, it is known that other pathogens cause skin lesions similar to those attributed to treponemes. In fact, the multiplex-PCR protocol [1] we applied has been developed on specimen from syphilis-like lesions, i.e. genital lesions. However, our study is the first to apply this multiplex-PCR approach on yaws-like lesions, even though *H. ducreyi* has also been reported in those lesions in the past. Out of the 156 tests performed, 132 were serologically negative and in only 23 of those was the presence of *T. pallidum*, *H. ducreyi*, HSV-1 and HSV-2 identified by amplification of their

DNA by multiplex-PCR approach (Tables 1 and 3). This leaves 109 serologically negative tests whose causative agent(s) remain(s) to be identified, although undetected *T. pallidum* DNA may be due to low sample quality (see Limitations section in the main text Discussion).

Despite the limitations discussed in the main text, our results emphasise that, with the recent developments of microbiome research, scientists cannot be thinking of skin ulcers as being caused by a single pathogen at a time, but should rather consider the community of related microbes that could interact to prevent healing. Including pathogens known to occur in similar skin ulcers will facilitate supporting communities that may be more at risk of being infected by those pathogens and will consequently move forwards achieving yaws eradication and syphilis elimination.

References

1. Glatz M, Juricevic N, Altwegg M, Bruisten S, Komericki P, Lautenschlager S, et al. A multicenter prospective trial to asses a new real-time polymerase chain reaction for detection of Treponema pallidum, herpes simplex-1/2 and Haemophilus ducreyi in genital, anal and oropharyngeal ulcers. Clin Microbiol Infect. 2014;20(12):O1020–7.
2. Boock AU, Awah PK, Mou F, Nichter M. Yaws resurgence in Bankim, Cameroon: The relative effectiveness of different means of detection in rural communities. PLoS neglected tropical diseases. 2017 May 8;11(5):e0005557.
3. Bhutta ZA, Sommerfeld J, Lassi ZS, Salam RA, Das JK. Global burden, distribution, and interventions for infectious diseases of poverty. Infect Dis poverty. 2014;3(1):1–7.
4. Mushayabasa S, Bhunu CP, Webb C, Dhlamini M. A mathematical model for assessing the impact of poverty on yaws eradication. Appl Math Model. 2012;36(4):1653–67.
5. Fitzpatrick C, Asiedu K, Jannin J. Where the road ends, yaws begins? The cost-effectiveness

of eradication versus more roads. PLoS Negl Trop Dis. 2014;8(9):e3165.

6. WHO. The global elimination of congenital syphilis: rationale and strategy for action [Internet]. 2008 [cited 2022 Jul 15]. Available from: https://www.who.int/publications/i/item/the-global-elimination-of-congenital-syphilis-rationale-and-strategy-for-action

7. Tsuboi M, Evans J, Davies EP, Rowley J, Korenromp EL, Clayton T, et al. Prevalence of syphilis among men who have sex with men: a global systematic review and meta-analysis from 2000-20. Lancet Glob Health. 2021 Aug;9(8):e1110-e1118.

8. Pětrošová H, Zobaníková M, Čejková D, Mikalová L, Pospíšilová P, Strouhal M, et al. Whole genome sequence of Treponema pallidum ssp. pallidum, strain Mexico A, suggests recombination between yaws and syphilis strains. PLoS Negl Trop Dis. 2012;6(9):e1832.

9. Arora N, Schuenemann VJ, Jäger G, Peltzer A, Seitz A, Herbig A, et al. Origin of modern syphilis and emergence of a pandemic Treponema pallidum cluster. Nat Microbiol. 2016;2(December):1–6.

10. Grillová L, Oppelt J, Mikalová L, Nováková M, Giacani L, Niesnerová A, et al. Directly Sequenced Genomes of Contemporary Strains of Syphilis Reveal Recombination-Driven Diversity in Genes Encoding Predicted Surface-Exposed Antigens. Front Microbiol. 2019;10:1691.

11. Noda AA, Méndez M, Rodríguez I, Šmajs D. Genetic recombination in Treponema pallidum: Implications for diagnosis, epidemiology, and vaccine development. Sex Transm Dis. 2022;49(1):e7–10.

12. Pla-Díaz M, Sánchez-Busó L, Giacani L, Šmajs D, Bosshard PP, Bagheri HC, et al. Evolutionary processes in the emergence and recent spread of the syphilis agent, Treponema pallidum. Mol Biol Evol. 2021;Epub-ahead.

13. Marks M, Fookes M, Wagner J, Butcher R, Ghinai R, Sokana O, et al. Diagnostics for Yaws Eradication: Insights From Direct Next-Generation Sequencing of Cutaneous Strains of Treponema pallidum. Clin Infect Dis. 2017/10/16. 2018 Mar 15;66(6):818–24.

14. Burke JP. International symposium on yaws and other endemic treponematoses. In: International Symposium on Yaws and Other Endemic Treponematoses (1984: Washington, DC). University of Chicago Press; 1985.

15. Csonka G, Pace J. Endemic nonvenereal treponematosis (bejel) in Saudi Arabia. Clinical Infectious Diseases. 1985 May 1;7(Supplement_2):S260-5.

16. Julvez J, Michault A, Kerdelhue V. Serologic studies of non-venereal treponematoses in infants in Niamey, Niger. Medecine Tropicale: Revue du Corps de Sante Colonial. 1998 Jan 1;58(1):38-40.

17. Čejková D, Zobanikova M, Chen L, Pospíšilová P, Strouhal M, Qin X, Mikalova L, Norris SJ, Muzny DM, Gibbs RA, Fulton LL. Whole genome sequences of three Treponema pallidum ssp. pertenue strains: yaws and syphilis treponemes differ in less than 0.2% of the genome sequence. PLoS neglected tropical diseases. 2012 Jan 24;6(1):e1471.
