# Supplementary material for: Prevalence of yaws and syphilis in the Ashanti region of Ghana and occurrence of H. ducreyi, herpes simplex virus 1 and herpes simplex virus 2 in skin lesions associated with treponematoses
Source: PLoS One. 2024 May 22;19(5):e0295088. doi: 10.1371/journal.pone.0295088 (PMC11111032; doi:10.1371/journal.pone.0295088)
Supplement: S4 File — (DOCX) [file pone.0295088.s007.docx]

References

1. Mitjà O, Marks M, Konan DJP, Ayelo G, Gonzalez-Beiras C, Boua B, et al. Global epidemiology of yaws: a systematic review. Lancet Glob Heal. 2015 May 19;3(6):e324–31.

2. Ayelo AG, Degboe BKE, Barogui YT, Gomido IC, Wadagni AC, D’Almeida C, et al. Resurgence of yaws in Benin: Four confirmed cases in the district of Z, Southern Benin. J Public Heal Epidemiol. 2019;11(9):201–8.

3. Herve V, Kassa EK, Normand P, Georges A, Mathiot C, Martin P. Resurgence of yaws in Central African Republic. Role of the Pygmy population as a reservoir of the virus. Bull Soc Pathol Exot. 1992;85(5):342–6.

4. Coldiron M, Obvala D, Mouniaman-Nara I, Pena J, Blondel C, Porten K. The prevalence of yaws among the Aka in the Congo. Med Sante Trop. 2013;23(2):231–2.

5. Gerstl S, Kiwila G, Dhorda M, Lonlas S, Myatt M, Ilunga BK, et al. Prevalence Study of Yaws in the Democratic Republic of Congo Using the Lot Quality Assurance Sampling Method (Prevalence of Yaws in DRC). Von Elm E, editor. PLoS One. 2009;4(7):e6338.

6. Konan YE, M’Bea KJ, Coulibaly A, Tetchi EO, Kpébo DO, Aké O, et al. A description of the yaws infection and prevention conditions in the health district of Adzopé. Sante Publique. 2007;19(2):111–8.

7. Anselmi M, Araujo E, Narvaez A, Cooper PJ, Guderian RH. Yaws in Ecuador: impact of control measures on the disease in the Province of Esmeraldas. Sex Transm Infect. 1995;71(6):343–6.

8. Anselmi M, Moreira J, Caicedo C, Guderian R, Tognoni G. Community participation eliminates yaws in Ecuador. Trop Med Int Heal. 2003;8(7):634–8.

9. Agana-Nsiire P, Kaitoo E, Agongo EEA, Bonsu G, Kyei-Faried S, Amponsa-Achiano K, et al. Yaws prevalence, lessons from the field and the way forward towards yaws eradication in Ghana. Int Sch Res Not. 2014;2014.

10. Abdulai AA, Agana-Nsiire P, Biney F, Kwakye-Maclean C, Kyei-Faried S, Amponsa-Achiano K, et al. Community-based mass treatment with azithromycin for the elimination of yaws in Ghana—Results of a pilot study. PLoS Negl Trop Dis. 2018;12(3):e0006303.

11. Basing LAW, Djan M, Simpson SV, Adu-Sarkodie Y. Mapping of yaws endemicity in Ghana; Lessons to strengthen the planning and implementation of yaws eradication. medRxiv. 2020;2020.02.20.20025122.

12. Scolnik D, Aronson L, Lovinsky R, Toledano K, Glazier R, Eisenstadt J, et al. Efficacy of a Targeted, Oral Penicillin—Based Yaws Control Program among Children Living in Rural South America. Clin Infect Dis. 2003;36(10):1232–8.

13. Noordhoek GT, Engelkens HJH, Judanarso J, Van Der Stek J, Aelbers GN, van der Sluis JJA, et al. Yaws in West Sumatra, Indonesia: clinical manifestations, serological findings and characterisation of new Treponema isolates by DNA probes. Eur J Clin Microbiol Infect Dis. 1991;10(1):12–9.

14. Sitanggang YA. Serologic observation and risk fact of yaws in Hamadi Public Health Center, Jayapura. Heal Sci J Indones. 2017;8(1).

15. Akogun OB. Yaws and Syphilis in the Garkida Area of Nigeria. Zentralblatt für Bakteriol. 1999;289(1):101–7.

16. Backhouse JL, Hudson BJ, Hamilton PA, Nesteroff SI. Failure of penicillin treatment of yaws on Karkar Island, Papua New Guinea. Backhouse JL, editor. Am J Trop Med Hyg. 1998;59(3):388–92.

17. Manning LA, Ogle GD. Yaws in the periurban settlements of Port Moresby, Papua New Guinea. Papua New Guinea Med J. 2002;45(3/4):206–12.

18. Mooring EQ, Mitjà O, Murray MB. Spatial-temporal clustering analysis of yaws on Lihir Island, Papua New Guinea to enhance planning and implementation of eradication programs. PLoS Negl Trop Dis. 2018;12(10):e0006840.

19. Ayove T, Houniei W, Wangnapi R, Bieb S V., Kazadi W, Luke LN, et al. Sensitivity and specificity of a rapid point-of-care test for active yaws: A comparative study. Lancet Glob Heal. 2014;2(7):e415–21.

20. Dofitas BL, Kalim SP, Toledo CB, Richardus JH. Yaws in the Philippines: first reported cases since the 1970s. Infect Dis poverty. 2020;9(1):1–16.

21. Marks M, Vahi V, Sokana O, Puiahi E, Pavluck A, Zhang Z, et al. Mapping the epidemiology of yaws in the Solomon Islands: a cluster randomized survey. Am J Trop Med Hyg. 2014/11/24. 2015 Jan;92(1):129–33.

22. Marks M, Goncalves A, Vahi V, Sokana O, Puiahi E, Zhang Z, et al. Evaluation of a rapid diagnostic test for yaws infection in a community surveillance setting. PLoS Negl Trop Dis. 2014 Sep 11;8(9):e3156–e3156.

23. dos Santos MML, Amaral S, Harmen SP, Joseph HM, Fernandes JL, Counahan ML. The prevalence of common skin infections in four districts in Timor-Leste: a cross sectional survey. BMC Infect Dis. 2010;10(1):61.

24. Harris M, Nako D, Hopkins T, Powell DM, Kenny C, Carroll C, et al. Yaws infection in Tanna, Vanuatu 1989. Southeast Asian J Trop Med Public Health. 1991;22(1):113–9.

25. Capuano C, Abel M. Campaign to eradicate yaws on Santo Island, Vanuatu in 2001. Med Trop Rev du Corps sante Colon. 2003;63(2):159–62.

26. Fegan D, Glennon MJ, Thami Y, Pakoa G. Resurgence of yaws in Tanna, Vanuatu: time for a new approach? Trop Doct. 2010;40(2):68–9.

27. Taleo F, Macleod CK, Marks M, Sokana O, Last A, Willis R, et al. Integrated mapping of yaws and trachoma in the five northern-most provinces of Vanuatu. PLoS Negl Trop Dis. 2017;11(1).

28. Guerrier G, Marcon S, Garnotel L, Deltour R, Schinas S, Mathelin JP, et al. Yaws in Polynesia’s Wallis and Futuna Islands: a seroprevalence survey. NZ Med J. 2011;124(1333):29–31.

29. Elyamany G, Al amro M, Costa Pereira W, Alsuhaibani O. Prevalence of Syphilis among Blood and Stem Cell Donors in Saudi Arabia: An Institutional Experience. Electron physician. 2016;8(8):2747–51.

30. Chen Y, Liu Z, Zhang Q, Chen J, Sun W, Yi J, et al. Trend in prevalence of syphilis among voluntary blood donors in Xi&#x2019;an, China from 2006 to 2010. Int J Infect Dis. 2014 Feb 1;19:98–9.

31. Drago F, Cogorno L, Ciccarese G, Strada P, Tognoni M, Rebora A, et al. Prevalence of syphilis among voluntary blood donors in Liguria region (Italy) from 2009 to 2013. Int J Infect Dis. 2014;28:e45–6.

32. Ekouevi DK, Bitty-Anderson AM, Gbeasor-Komlanvi FA, Konu YR, Sewu EK, Salou M, et al. Low prevalence of syphilis infection among key populations in Togo in 2017: a national cross-sectional survey. Arch Public Health. 2019 Sep 5;77:39.

33. Halatoko WA, Landoh DE, Saka B, Akolly K, Layibo Y, Yaya I, et al. Prevalence of syphilis among female sex workers and their clients in Togo in 2011. BMC Public Health. 2017;17(1):219.

34. Liao M, Nie X, Pan R, Wang C, Ruan S, Zhang C, et al. Consistently low prevalence of syphilis among female sex workers in Jinan, China: Findings from two consecutive respondent driven sampling surveys. PLoS One. 2012;7(4).

35. Chen X-S, Wang Q-Q, Yin Y-P, Liang G-J, Jiang N, Yang L-G, et al. Prevalence of syphilis infection in different tiers of female sex workers in China: implications for surveillance and interventions. BMC Infect Dis. 2012 Apr 4;12:84.

36. Tao XH, Jiang T, Shao D, Xue W, Ye FS, Wang M, et al. High prevalence of syphilis among street-based female sex workers in Nanchang, China. Indian Dermatol Online J. 2014 Oct;5(4):449–55.

37. Borges BVDS, Oliveira VMC De, Brito GMI, Gir E, Galvão MTG, Magalhães RLB De. Lb3.252 Prevalence of syphilis and factors associated with femake sex workers in teresina, piauÍ. Vol. 93, Sexually Transmitted Infections. BMJ Publishing Group Ltd; 2017. p. A187.

38. Oukouchoud H, Ouanaim C, Bellaji B, Hançali A, Jennane S, Bennani A, et al. P3.176 Sero-prevalence of syphilis among female sex workers in morocco. Vol. 93, Sexually Transmitted Infections. BMJ Publishing Group Ltd; 2017. p. A158.

39. Ferreira-Júnior ODC, Guimarães MDC, Damacena GN, de Almeida WDS, de Souza-Júnior PRB, Szwarcwald CL. Prevalence estimates of HIV, syphilis, hepatitis B and C among female sex workers (FSW) in Brazil, 2016. Medicine (Baltimore). 2018;97(1S Suppl 1):S3.

40. Cunha C, Friedman R, de Boni R, Gaydos C, Guimaraes M, Siqueira B, et al. Chlamydia trachomatis, Neisseria gonorrhoeae and syphilis among men who have sex with men in Brazil. BMC Public Health. 2015;15:n/a.

41. Coelho HC, Passos ADC. Low prevalence of syphilis in Brazilian inmates. Brazilian J Infect Dis. 2011 Feb;15(1):94–5.

42. Kazi AM, Shah SA, Jenkins CA, Shepherd BE, Vermund SH. Risk factors and prevalence of tuberculosis, human immunodeficiency virus, syphilis, hepatitis B virus, and hepatitis C virus among prisoners in Pakistan. Int J Infect Dis. 2010;14:e60–6.

43. Miranda AE, Vargas PM, Louis MEST, VIANA MC. Sexually transmitted diseases among female prisoners in Brazil: prevalence and risk factors. Sex Transm Dis. 2000;27(9):491–5.

44. Azbel L, Polonsky M, Wegman M, Shumskaya N, Kurmanalieva A, Asanov A, et al. Intersecting epidemics of HIV, HCV, and syphilis among soon-to-be released prisoners in Kyrgyzstan: Implications for prevention and treatment. Int J Drug Policy. 2016;37:9–20.

45. Adjei AA, Armah HB, Gbagbo F, Ampofo WK, Quaye IKE, Hesse IFA, et al. Prevalence of human immunodeficiency virus, hepatitis B virus, hepatitis C virus and syphilis among prison inmates and officers at Nsawam and Accra, Ghana. J Med Microbiol. 2006;55(5):593–7.

46. Jun-Jie X, Reilly K, Jing Z, Yong-Jun J, Hong S. P1-184 A meta-analysis of the prevalence of HIV/syphilis among Chinese bisexual men who have sex with men (MSM). Jun-Jie X, editor. J Epidemiol Community Health. 2011;65:A117–A117.

47. Heffelfinger JD, Swint EB, Berman SM, Weinstock HS. Trends in primary and secondary syphilis among men who have sex with men in the United States. Am J Public Health. 2007;97(6):1076–83.

48. Hernandez I, Johnson A, Reina-Ortiz M, Rosas C, Sharma V, Teran S, et al. Syphilis and HIV/Syphilis Co-infection Among Men Who Have Sex With Men (MSM) in Ecuador. Am J Mens Health. 2017;11(4):823–33.

49. Johnston LG, Alami K, El Rhilani MH, Karkouri M, Mellouk O, Abadie A, et al. HIV, syphilis and sexual risk behaviours among men who have sex with men in Agadir and Marrakesh, Morocco. Vol. 89, Sexually Transmitted Infections. BMJ Publishing Group Ltd; 2013. p. iii45.

50. Domingues RMSM, Leal M do C, Pereira APE, Ayres B, Sánchez AR, Larouzé B. Prevalence of syphilis and HIV infection during pregnancy in incarcerated women and the incidence of congenital syphilis in births in prison in Brazil. Cad Saude Publica. 2017;33(11):e00183616.

51. Vu L, Misra K. High Burden of HIV, Syphilis and HSV-2 and Factors Associated with HIV Infection Among Female Sex Workers in Tanzania: Implications for Early Treatment of HIV and Pre-exposure Prophylaxis (PrEP). AIDS Behav. 2018;22(4):1113–21.

52. Mutagoma M, Nyirazinyoye L, Sebuhoro D, Riedel DJ, Ntaganira J. Syphilis and HIV prevalence and associated factors to their co-infection, hepatitis B and hepatitis C viruses prevalence among female sex workers in Rwanda. BMC Infect Dis. 2017;17(1):525–9.

53. Chen Y, Shen Z, Morano JP, Khoshnood K, Wu Z, Lan G, et al. Bridging the epidemic: a comprehensive analysis of prevalence and correlates of HIV, hepatitis C, and syphilis, and infection among female sex workers in Guangxi Province, China. PLoS One. 2015;10(2):e0115311.

54. Zhou C, Rou K, Dong WM, Wang Y, Dong W, Zhou Y, et al. High prevalence of HIV and syphilis and associated factors among low-fee female sex workers in mainland China: a cross-sectional study. BMC Infect Dis. 2014;14(1):225.

55. Cárcamo CP, Campos PE, García PJ, Hughes JP, Garnett GP, Holmes KK. Prevalences of sexually transmitted infections in young adults and female sex workers in Peru: a national population-based survey. Lancet Infect Dis. 2012;12(10):765–73.

56. Majid N, Bollen L, Morineau G, Daily SF, Mustikawati DE, Agus N, et al. Syphilis among female sex workers in Indonesia: need and opportunity for intervention. Sex Transm Infect. 2010;86(5):377–83.

57. Kakchapati S, Singh DR, Rawal BB, Lim A. Sexual risk behaviors, HIV, and syphilis among female sex workers in Nepal. HIV AIDS (Auckl). 2017 Jan 27;9:9–18.

58. Mejia A, Bautista CT, Leal L, Ayala C, Prieto F, de la Hoz F, et al. Syphilis infection among female sex workers in Colombia. J Immigr Minor Heal. 2009;11(2):92–8.

59. Pando MA, Berini C, Bibini M, Fernández M, Reinaga E, Maulen S, et al. Prevalence of HIV and other sexually transmitted infections among female commercial sex workers in Argentina. Am J Trop Med Hyg. 2006;74(2):233–8.

60. Ouedraogo HG, Meda IB, Zongo I, Ky-Zerbo O, Grosso A, Samadoulougou BC, et al. Syphilis among female sex workers: results of point-of-care screening during a cross-sectional behavioral survey in Burkina Faso, West Africa. Int J Microbiol. 2018;2018.

61. Choi K-H, Ning Z, Gregorich SE, Pan Q. The influence of social and sexual networks in the spread of HIV and syphilis among men who have sex with men in Shanghai, China. JAIDS J Acquir Immune Defic Syndr. 2007;45(1):77–84.

62. Wu Z, Xu J, Liu E, Mao Y, Xiao Y, Sun X, et al. HIV and Syphilis Prevalence Among Men Who Have Sex With Men: A Cross-Sectional Survey of 61 Cities in China. Clin Infect Dis. 2013;57(2):298–309.

63. Wang X, Lan G, Shen Z, Vermund SH, Zhu Q, Chen Y, et al. HIV and syphilis prevalence trends among men who have sex with men in Guangxi, China: yearly cross-sectional surveys, 2008-2012. BMC Infect Dis. 2014;14(1):367.

64. Ouedraogo HG, Zida S, Compaore TR, Lanou BH, Rao A, Sagna T, et al. Seroepidemiology of syphilis among men who have sex with men in Burkina Faso, West Africa. Eur J Clin Microbiol Infect Dis. 2019;38(10):1803–9.

65. Hakre S, Arteaga GB, Núñez AE, Arambu N, Aumakhan B, Liu M, et al. Prevalence of HIV, Syphilis, and Other Sexually Transmitted Infections among MSM from Three Cities in Panama. J Urban Heal. 2014;91(4):793–808.

66. Zorzi A, Cordioli M, Gios L, Del Bravo P, Toskin I, Peeling RW, et al. Field evaluation of two point-of-care tests for syphilis among men who have sex with men, Verona, Italy. Sex Transm Infect. 2017;93(S4):S51–8.

67. Storm M, Deuba K, Damas J, Shrestha U, Rawal B, Bhattarai R, et al. Prevalence of HIV, syphilis, and assessment of the social and structural determinants of sexual risk behaviour and health service utilisation among MSM and transgender women in Terai highway districts of Nepal: findings based on an integrated biological a. BMC Infect Dis. 2020;20(1):1–14.

68. Prado, I., Redoschi, B. R. L., Welikow, A., Wilson, E. C., Turner, C. M. Syphilis prevalence and hiv co-infection amongst men who have sex with men (MSM) in sao paulo, 2011 and 2016. Sex Transm Infect. 2017;93(Suppl 2):A129.

69. Pisani E, Girault P, Gultom M, Sukartini N, Kumalawati J, Jazan S, et al. HIV, syphilis infection, and sexual practices among transgenders, male sex workers, and other men who have sex with men in Jakarta, Indonesia. Sex Transm Infect. 2004;80(6):536–40.

70. Adjei AA, Kudzi W, Armah H, Adiku T, Amoah AGB, Ansah J. Prevalence of antibodies to syphilis among blood donors in Accra, Ghana. Jpn J Infect Dis. 2003;56(4):165–7.

71. Matee MIN, Magesa PM, Lyamuya EF. Seroprevalence of human immunodeficiency virus, hepatitis B and C viruses and syphilis infections among blood donors at the Muhimbili National Hospital in Dar Es Salaam, Tanzania. BMC Public Health. 2006;6(1):21.

72. Nagalo MB, Sanou M, Bisseye C, Kaboré MI, Nebie YK, Kienou K, et al. Seroprevalence of human immunodeficiency virus, hepatitis B and C viruses and syphilis among blood donors in Koudougou (Burkina Faso) in 2009. Blood Transfus. 2011/07/18. 2011 Oct;9(4):419–24.

73. Diarra A, Kouriba B, Baby M, Murphy E, Lefrere J-J. HIV, HCV, HBV and syphilis rate of positive donations among blood donations in Mali: Lower rates among volunteer blood donors. Transfus Clin Biol. 2009;16(5):444–7.

74. Shrestha AC, Ghimire P, Tiwari BR, Rajkarnikar M. Transfusion-transmissible infections among blood donors in Kathmandu, Nepal. J Infect Dev Ctries. 2009;3(10):794–7.

75. Damulak OD, Jatau E, Akinga E, Peter G. The prevalence of syphilis among blood donors in a centralized Nigerian Blood Transfusion Service Centre. Niger J Med. 2013;22(2):113–6.

76. Buseri FI, Muhibi MA, Jeremiah ZA. Sero-epidemiology of transfusion-transmissible infectious diseases among blood donors in Osogbo, south-west Nigeria. Blood Transfus. 2009;7(4):293.

77. Bhawani Y, Rao PR, Sudhakar V. Seroprevalence of transfusion transmissible infections among blood donors in a tertiary care hospital of Andhra Pradesh. Biol Med. 2010;2(4):45–8.

78. Elfaki AMH, Eldour AA, Elsheikh NMH. Sero-prevalence of immunodeficiency virus, hepatitis B and C and syphilis among blood donors at ElObeid Teaching Hospital, West Sudan. Sudan J Med Sci. 2008;3(4):333–8.

79. Abate M, Wolde T. Seroprevalence of human immunodeficiency virus, hepatitis B virus, hepatitis C virus, and syphilis among blood donors at jigjiga blood bank, eastern Ethiopia. Ethiop J Health Sci. 2016;26(2):155–62.

80. Olokoba AB, Olokoba LB, Salawu FK, Danburam A, Desalu OO, Badung LH, et al. Syphilis in voluntary blood donors in North-Eastern Nigeria. Eur J Sci Res. 2009;31(3):335–40.

81. Sarkodie F, Hassall O, Owusu-Dabo E, Owusu-Ofori S, Bates I, Bygbjerg IC, et al. Syphilis screening practices in blood transfusion facilities in Ghana. Int J Infect Dis. 2016 Feb 1;43:90–4.

82. Solomon L, Flynn C, Muck K, Vertefeuille J. Prevalence of HIV, syphilis, hepatitis B, and hepatitis C among entrants to Maryland correctional facilities. J Urban Heal. 2004;81(1):25–37.

83. El Maerrawi I, Carvalho HB. Prevalence and risk factors associated with HIV infection, hepatitis and syphilis in a state prison of São Paulo. Int J STD AIDS. 2015;26(2):120–7.

84. Waheed U, Satti HS, Arshad M, Farooq A, Rauf A, Zaheer HA. Epidemiology of HIV/AIDS and Syphilis among high risk groups in Pakistan. Pak J Zool. 2017;49(5).

85. Baillargeon J, Black SA, Leach CT, Jenson H, Pulvino J, Bradshaw P, et al. The infectious disease profile of Texas prison inmates. Prev Med (Baltim). 2004;38(5):607–12.

86. Catalan-Soares BC, Almeida RTP, Carneiro-Proietti ABF. Prevalence of HIV-1/2, HTLV-I/II, hepatitis B virus (HBV), hepatitis C virus (HCV), Treponema pallidum and Trypanosoma cruzi among prison inmates at Manhuaçu, Minas Gerais State, Brazil. Rev Soc Bras Med Trop. 2000;33(1):27–30.

87. Correa ME, Croda J, Coimbra Motta de Castro AR, Maria do Valle Leone de Oliveira S, Pompilio MA, Omizolo de Souza R, et al. High Prevalence of Treponema pallidum Infection in Brazilian Prisoners. Am J Trop Med Hyg. 2017/07/31. 2017 Oct 11;97(4):1078–84.

88. Rhodes T, Platt L, Maximova S, Koshkina E, Latishevskaya N, Hickman M, et al. Prevalence of HIV, hepatitis C and syphilis among injecting drug users in Russia: a multi‐city study. Addiction. 2006;101(2):252–66.

89. Scherbaum N, Baune BT, Mikolajczyk R, Kuhlmann T, Reymann G, Reker M. Prevalence and risk factors of syphilis infection among drug addicts. BMC Infect Dis. 2005;5(1):33.
