# Supplementary material for: Prevalence of yaws and syphilis in the Ashanti region of Ghana and occurrence of H. ducreyi, herpes simplex virus 1 and herpes simplex virus 2 in skin lesions associated with treponematoses
Source: PLoS One. 2024 May 22;19(5):e0295088. doi: 10.1371/journal.pone.0295088 (PMC11111032; doi:10.1371/journal.pone.0295088)
Supplement: Attachment — Submitted filename: 2023_YawsGhana_comments_HZ_def.docx [file pone.0295088.s008.docx]

GENERAL COMMENTS:

The manuscript titled “Prevalence of yaws and syphilis in the Ashanti region of Ghana and occurrence of H.ducreyi, Herpes simplex virus 1 and Herpes simplex virus 2 in skin lesions associated with treponematoses” by Agyekum et al. presents important results regarding the prevalence of *T. pallidum* and occurrence of different pathogens in skin lesions that are associated with treponematoses. While the manuscript contains interesting data that has a significant value to the research field, the manuscript has a lot of parts that are too detailed. This makes it harder to follow and takes away from the main results and aim of the work. My general comments and specific suggestions can be found below.

General comments

- Subspecies should be in lower case “*Treponema pallidum* subsp. *pallidum"*.
- The overall manuscript needs to be more concise.
- The use of references is overabundant and can be greatly reduced in number.
- What is the reason that *Treponema pallidum* subspecies *endemicum* is not mentioned at all in the manuscript? The endemic areas of both diseases are very close and lesions may also occur in bejel. Please explain.

Introduction

1. Figure 1 shows the results of a literature study as described in the figure legend. However, if considered relevant for the context of the results, this should be included in the method section and the figure moved to the results.

Material and Methods

1. Line 141 – “Participants with syphilis” instead of “Syphilis participants”.
2. Include information on participants with yaws-like lesions (as mentioned in legend of Figure 2).
3. Lines 159 – 160 – “Participants from 5-17 years” add “of age” or rephrase.
4. Line 161 – Remove ‘from’
5. Line 165- define clinical diagnoses, is this based on age and clinical manifestations only? Are the participants asked about their sexual behavior/activity?
6. Line 180 – add ‘by’ before ‘Glatz’
7. What additional data was collected from the participants? In the results data is presented on ethnicity. Please add in the method section.

Results

1. The information presented in figures 3, 4 and 5 can be summarized in one table including statistical results. Also the demographics on age and sex as described in the first paragraph can be included.
2. Line 217 – “Participants were asked to self-define their ethnicity.” Move this information to the method section.
3. After mentioning that the ethnicity data comes from self-defining, this does not need to be repeated throughout the manuscript.
4. If the confidence intervals are considered relevant, please add this information to the summarized table as suggested earlier or, if the authors disagree with the suggestion, add this to figure 3.
5. The paragraph on seroprevalence only show percentages, please add full numbers as well.
6. The pairwise analysis of seroprevalence per region may be easier to follow by using a heatmap-like table or figure in order for the readers to have an overview of what is being explained.

Discussion

1. The discussion section is too long (10 pages) and should be shortened to half its size.
2. The serological assay was positive in a low percentage of the total suspected lesions of yaws and syphilis. What was the cause of the lesions if not these pathogens?
3. Lines 354 – 361 – The paragraph on congenital syphilis may be removed. The manuscript provides important data on prevalences, but its relevance to the elimination of congenital syphilis can be mentioned in one sentence.
4. I agree that the co-occurrence of syphilis and yaws in the same geographical area is interesting. Paragraph 362 – 379 should include information on *Treponema pallidum* subsp *endemicum.*
5. Paragraph on recombination should be removed as this is outside of the scope of this manuscript.
6. The limitation section could be more extensive including limitations in sample size per location, limited behavioral data, the use of a multiplex PCR with possibly less sensitive results?
7. Looking at the seroprevalence, misdiagnoses of syphilis and yaws seem to be very common as other ulcer-causing pathogen were detected instead of *T. pallidum.* Only 17.2% of the yaws-like lesions and 10.8% of the syphilis-like lesions were positive by serological testing (and one yaws-like lesions by PCR). This would suggest that 82.8% and 89.2% are not yaws or syphilis and may thereby have been misdiagnosed. If that is correct then please rephrase 522 – 524 “The prevalence … lesions determined.” Change “co-occurrence” into “occurrence”.
