# Supplementary material for: Prevalence of yaws and syphilis in the Ashanti region of Ghana and occurrence of H. ducreyi, herpes simplex virus 1 and herpes simplex virus 2 in skin lesions associated with treponematoses
Source: PLoS One. 2024 May 22;19(5):e0295088. doi: 10.1371/journal.pone.0295088 (PMC11111032; doi:10.1371/journal.pone.0295088)
Supplement: Attachment — Submitted filename: Boaitey_et_al_ResponseToReviewers2024.03.08.docx [file pone.0295088.s009.docx]

PONE-D-23-37271- REBUTTAL LETTER -

Prevalence of yaws and syphilis in the Ashanti region of Ghana and occurrence of H. ducreyi, Herpes simplex virus 1 and Herpes simplex virus 2 in skin lesions associated with treponematoses.

PLOS ONE

Dear PLOS ONE Academic Editor Dr Sylvia Maria Bruisten,

We thank you and the two reviewers who have provided comments on our manuscript for considering our manuscript PONE-D-23-37271 for publication in PLOS ONE. This is a rebuttal letter that responds to each point raised by the academic editor and both reviewers. These points have been left in black font below. Our responses to each of those points is in blue font. When we cite sections of our revised manuscript, the text appears in black and italic font.

Journal requirements:

1. When submitting your revision, we need you to address these additional requirements.

Please ensure that your manuscript meets PLOS ONE's style requirements, including those for file naming. The PLOS ONE style templates can be found at 
https://journals.plos.org/plosone/s/file?id=wjVg/PLOSOne_formatting_sample_main_body.pdf and 
https://journals.plos.org/plosone/s/file?id=ba62/PLOSOne_formatting_sample_title_authors_affiliations.pdf.

We have modified the style of our manuscript so that level 1 headings read in font size 18pt and level 2 headings read in font size 16pt. We have also changed the word “Figure” for “Fig” when referring to one of the manuscript’s Figures. Tables have been formatted according to the PLOS ONE style template available online.

On the title page, author surnames are in small letters, apart from the first letter, which is a capital letter. Numbers referring to author’s affiliations are all in superscript. The corresponding author’s name has an asterisk superscript and their email address is provided, with their initials in parenthesis next to it.

2. Please include a complete copy of PLOS’ questionnaire on inclusivity in global research in your revised manuscript. Our policy for research in this area aims to improve transparency in the reporting of research performed outside of researchers’ own country or community. The policy applies to researchers who have travelled to a different country to conduct research, research with Indigenous populations or their lands, and research on cultural artefacts. The questionnaire can also be requested at the journal’s discretion for any other submissions, even if these conditions are not met.  Please find more information on the policy and a link to download a blank copy of the questionnaire here: https://journals.plos.org/plosone/s/best-practices-in-research-reporting. Please upload a completed version of your questionnaire as Supporting Information when you resubmit your manuscript.

We have completed PLOS’ questionnaire on inclusivity in global research and have included this file in the Supporting Information (S1 File). In addition, a section “Inclusivity in global research” has been added in the Methods section with the sentence “Additional information regarding the ethical, cultural, and scientific considerations specific to inclusivity in global research is included in the Supporting Information (S1 File)”.

3. Thank you for stating the following in the Acknowledgments Section of your manuscript: 
[We thank the Global Research Challenge Fund - University of Westminster grant number SRO03150 (PG, SR) for funding this research. We also thank the Royal Society and the Bill and Melinda Gates Foundation for funding molecular work and training, respectively. We thank Dr Patrick Kimmitt for comments on earlier version of this manuscript and Kostas Kampourakis.
This work was supported, in part, by the Bill & Melinda Gates Foundation [INV-036560]. Under the grant conditions of the Foundation, a Creative Commons Attribution 4.0 Generic License has already been assigned to the Author Accepted Manuscript version that might arise from this submission.]
We note that you have provided funding information that is not currently declared in your Funding Statement. However, funding information should not appear in the Acknowledgments section or other areas of your manuscript. We will only publish funding information present in the Funding Statement section of the online submission form. 
Please remove any funding-related text from the manuscript and let us know how you would like to update your Funding Statement. Currently, your Funding Statement reads as follows: 
 [Global Research Challenge Fund - University of Westminster grant number SRO03150 (PG, SR)
Bill and Melinda Gates Foundation grant number INV-036560 (JBP)
Royal Society International Exchanges 2021 Round 3 award number IES\R3\213065 (SR, PPB)
None of the sponsors or funders played any role in the study design, data collection and analysis, decision to publish, or preparation of the manuscript.]

Please include your amended statements within your cover letter; we will change the online submission form on your behalf.

We have removed the funding information from the Acknowledgments Section, which now reads as follows: “*We thank Dr Patrick Kimmitt, Kostas Kampourakis, Helene Zondag and one anonymous Reviewer for comments on earlier versions of this manuscript. We are also grateful to Nicole Zimmermann for her work on the multiplex-PCR.*”

We would like to modify the funding information in the Funding Statement section online to read as follows:

“YAB research was funded by a PhD studentship from the Global Research Challenge Fund - University of Westminster, grant number SRO03150 (PG, SR).

This work was supported, in part, by the Bill and Melinda Gates Foundation, grant number INV-036560 (JBP). Under the grant conditions of the Foundation, a Creative Commons Attribution 4.0 Generic License has already been assigned to the Author Accepted Manuscript version that might arise from this submission.

This work was also partly supported by a Royal Society International Exchanges 2021 Round 3, award number IES\R3\213065 (SR, PPB).

None of the sponsors or funders played any role in the study design, data collection and analysis, decision to publish, or preparation of the manuscript.”

4. Thank you for stating the following in the Competing Interests section: 
[JBP reports research support from Gilead Sciences, non-financial support from Abbott Laboratories, and consulting for Zymeron Corporation, all outside the scope of this work. All other authors declare no competing interests].

Please confirm that this does not alter your adherence to all PLOS ONE policies on sharing data and materials, by including the following statement: ""This does not alter our adherence to  PLOS ONE policies on sharing data and materials.” (as detailed online in our guide for authors http://journals.plos.org/plosone/s/competing-interests).  If there are restrictions on sharing of data and/or materials, please state these. Please note that we cannot proceed with consideration of your article until this information has been declared.

Please include your updated Competing Interests statement in your cover letter; we will change the online submission form on your behalf.

We have removed the Competing interests section from our manuscript and we confirm that the competing interests declared does not alter our adherence to PLOS ONE policies on sharing data and materials. Please update our online submission form so that the Competing Interests statement reads as follows:

“JBP reports research support from Gilead Sciences, non-financial support from Abbott Laboratories, and consulting for Zymeron Corporation, all outside the scope of this work. This does not alter our adherence to PLOS ONE policies on sharing data and materials. All other authors declare no competing interests.”

5. We note that Figure 1B, 1C and 2 in your submission contain [map/satellite] images which may be copyrighted. All PLOS content is published under the Creative Commons Attribution License (CC BY 4.0), which means that the manuscript, images, and Supporting Information files will be freely available online, and any third party is permitted to access, download, copy, distribute, and use these materials in any way, even commercially, with proper attribution. For these reasons, we cannot publish previously copyrighted maps or satellite images created using proprietary data, such as Google software (Google Maps, Street View, and Earth). For more information, see our copyright guidelines: http://journals.plos.org/plosone/s/licenses-and-copyright.
 
We require you to either (1) present written permission from the copyright holder to publish these figures specifically under the CC BY 4.0 license, or (2) remove the figures from your submission:
 
a. You may seek permission from the original copyright holder of Figure 1B, 1C and 2 to publish the content specifically under the CC BY 4.0 license.  
 
We recommend that you contact the original copyright holder with the Content Permission Form (http://journals.plos.org/plosone/s/file?id=7c09/content-permission-form.pdf) and the following text:
“I request permission for the open-access journal PLOS ONE to publish XXX under the Creative Commons Attribution License (CCAL) CC BY 4.0 (http://creativecommons.org/licenses/by/4.0/). Please be aware that this license allows unrestricted use and distribution, even commercially, by third parties. Please reply and provide explicit written permission to publish XXX under a CC BY license and complete the attached form.”
 
Please upload the completed Content Permission Form or other proof of granted permissions as an ""Other"" file with your submission.
 
In the figure caption of the copyrighted figure, please include the following text: “Reprinted from [ref] under a CC BY license, with permission from [name of publisher], original copyright [original copyright year].”
 
b. If you are unable to obtain permission from the original copyright holder to publish these figures under the CC BY 4.0 license or if the copyright holder’s requirements are incompatible with the CC BY 4.0 license, please either i) remove the figure or ii) supply a replacement figure that complies with the CC BY 4.0 license. Please check copyright information on all replacement figures and update the figure caption with source information. If applicable, please specify in the figure caption text when a figure is similar but not identical to the original image and is therefore for illustrative purposes only.
The following resources for replacing copyrighted map figures may be helpful:
 
USGS National Map Viewer (public domain): http://viewer.nationalmap.gov/viewer/
The Gateway to Astronaut Photography of Earth (public domain): http://eol.jsc.nasa.gov/sseop/clickmap/
Maps at the CIA (public domain): https://www.cia.gov/library/publications/the-world-factbook/index.html and https://www.cia.gov/library/publications/cia-maps-publications/index.html
NASA Earth Observatory (public domain): http://earthobservatory.nasa.gov/
Landsat: http://landsat.visibleearth.nasa.gov/
USGS EROS (Earth Resources Observatory and Science (EROS) Center) (public domain): http://eros.usgs.gov/#
Natural Earth (public domain): <http://www.naturalearthdata.com/>

This is a misunderstanding, we apologise, as this information was not clear from our initial description of the methods. The maps shown on Figures 1B, 1C and 2A, 2B and 2C were all produced using R packages, including ggplot2, rworldmap, maps, raster, countrycode, viridis, scales, dplyr, cowplot. Because we produced those maps, no copyright permission needs to be sought. We have edited one of the sentences in the Statistical analysis subsection of the Methods section (Statistical analysis, page 8, lines 170-174) of the revised manuscript, so that it now reads:

“*Figures were produced with the ggplot2 package (version 3.4.0) and maps were obtained using additional R packages, including rworldmap, maps, raster, countrycode, viridis, scales, dplyr, cowplot. R scripts used to produce the manuscript’s Figures, Supporting Information Figures and statistical analyses are included in the Supporting Information (S2 File).* ”

We have added this file in the Supporting information file list and uploaded the file.

6. Please include captions for your Supporting Information files at the end of your manuscript, and update any in-text citations to match accordingly. Please see our Supporting Information guidelines for more information: http://journals.plos.org/plosone/s/supporting-information.

A section called Supporting Information, listing additional files and Figures, has been added at the end of the manuscript pages 31- 32, lines 662-692. This reads as follows:

*“Supporting Information*

*S1 Fig. Prevalence of yaws and syphilis estimated from studies published between 1991 and 2020. (A) Prevalence of yaws and syphilis estimated from 89 peer-reviewed studies (number of positive cases for yaws or syphilis divided by the total number of participants per study). Out of the 15 countries known to be endemic for yaws, 13 are represented in the selected peer-reviewed articles reporting the prevalence of yaws or syphilis (no data published between 1991 and 2020 on the prevalence of yaws and syphilis from Cameroon and Liberia that fitted our criteria). Only four countries showed estimates of the prevalence of yaws and syphilis, from independent studies, i.e. Ecuador, Ghana, Indonesia, Nigeria. For comparison, blue triangles show the seroprevalence of yaws and syphilis estimated in the Ashanti region of Ghana from this study. There is no significant difference between any of the estimated prevalence of yaws and syphilis (Wilcoxon Rank Sum test p-values are 0.77 between the published prevalence of yaws and syphilis (globally); 0.67 between the published prevalence of yaws and syphilis in Ecuador; 0.70 between the published prevalence of yaws and syphilis in Ghana; 1 between the published prevalence of yaws and syphilis in Indonesia; 0.50 between the published prevalence of yaws and syphilis in Nigeria. The Chi-squared test p-value is 0.44 between the observed seroprevalence of yaws and syphilis in the Ashanti region). (B) Median of the estimated prevalence of yaws by country in studies published between 1991 and 2020. The borders of the four countries where the prevalence of both yaws and syphilis have been reported (Ecuador, Ghana, Indonesia, Nigeria) are represented in red. (C) Median of the estimated prevalence of syphilis by country in studies published between 1991 and 2020. The borders of the four countries where the prevalence of both yaws and syphilis have been reported (Ecuador, Ghana, Indonesia, Nigeria) are represented in red.*

*S1 Table. Data collected from the 156 participants from the Ashanti region of Ghana, this study.*

*S2 Table. Data from 89 peer-reviewed articles reporting the prevalence of yaws and syphilis.*

*S1 File. Inclusivity in global research questionnaire.*

*S2 File. Rscript for reproducing data analyses and graphs, including maps.*

*S3 File. Extensive discussion with references.*

*S4 File. References for the 89 peer-reviewed research articles in S2 Table.”*

Additional Editor Comments:

Both reviewers have good comments with which I fully agree. 

We thank both reviewers for their comments. We address them point by point in our response below. The Reviewers’ point reads in black font and our response in blue. Text cited from the revised manuscript appears in black and italic font.

5. Review Comments to the Author

Reviewer #1: GENERAL COMMENTS:
The manuscript titled “Prevalence of yaws and syphilis in the Ashanti region of Ghana and occurrence of H.ducreyi, Herpes simplex virus 1 and Herpes simplex virus 2 in skin lesions associated with treponematoses” by Agyekum et al. presents important results regarding the prevalence of T. pallidum and occurrence of different pathogens in skin lesions that are associated with treponematoses. While the manuscript contains interesting data that has a significant value to the research field, the manuscript has a lot of parts that are too detailed. This makes it harder to follow and takes away from the main results and aim of the work. My general comments and specific suggestions can be found below.

General comments
- Subspecies should be in lower case “Treponema pallidum subsp. pallidum”. This has been addressed throughout the manuscript.
- The overall manuscript needs to be more concise. We have made an effort to shorten the manuscript. It is currently 24 pages from the Title page to the Acknowledgments, including two additional Tables that were not shown in the original version of the manuscript (which was 25 pages long from the Title page to the Acknowledgments).
- The use of references is overabundant and can be greatly reduced in number. There were 79 references in the original version of the manuscript, there are now 56 references.
- What is the reason that Treponema pallidum subspecies endemicum is not mentioned at all in the manuscript? The endemic areas of both diseases are very close and lesions may also occur in bejel. Please explain. This has been addressed and further explained below as this is another point raised by Reviewer#1 below.

Introduction
1. Figure 1 shows the results of a literature study as described in the figure legend. However, if considered relevant for the context of the results, this should be included in the method section and the figure moved to the results.

We appreciate this comment and, in our revised version, we have moved this Figure to the Supplementary Information (S1 Fig). Moving this Figure and the information associated to it also contributes to making the overall manuscript more concise (one of Reviewer #1 General comments). Files associated to this Figure are listed in the Supplementary Information as *S1 Fig. Prevalence of yaws and syphilis estimated from studies published between 1991 and 2020*.

Material and Methods
1. Line 141 – “Participants with syphilis” instead of “Syphilis participants”.

Thank you for pointing this out. This has actually been worded more specifically as “*Participants with syphilis-like lesions were recruited…*” page 5 line 95 (Methods, Study communities) in the revised version of the manuscript.

2. Include information on participants with yaws-like lesions (as mentioned in legend of Figure 2).

Please note that Figure 2 of our initial submission now becomes Fig 1 in our revised version of the manuscript. The information about participants with yaws-like lesions has now been added page 5 lines 91-95 (Methods, Study communities) and reads as follows (as it appears in the Fig 1 legend): “*Participants with yaws-like lesions were recruited from three locations: Afigya Kwabre South municipality (Afigya), with study sites Adweratia and Mpobi ; Juaben municipality, with study sites Kotei and Atia ; and Atwima Kwanwoma municipality (Atwima), with study sites Brofoyedu and Bebu.* ”

3. Lines 159 – 160 – “Participants from 5-17 years” add “of age” or rephrase
4. Line 161 – Remove ‘from’

These lines 159-161 are now page 5-6 lines 113-116 (Methods, Sample and data collection) of the revised version of the manuscript and the corresponding sentences have been modified so that they now read: “*Participants from 5-17 years of age gave their verbal assent before their guardians were asked to sign the informed consent form. Participants who were 5 to 17 years of age with a minimum of one clinical lesion consistent with primary or secondary treponemal (yaws) infection [2] were enrolled for yaws-like lesion samples.*”

5. Line 165- define clinical diagnoses, is this based on age and clinical manifestations only? Are the participants asked about their sexual behavior/activity?

We have added a section in our Methods page 6, lines 129-131 of the revised version of the manuscript called “*Clinical diagnosis and seroprevalence*”, where we have rephrased the sentence: “*Clinical diagnoses of yaws- and syphilis-like lesions by study clinician and a trained community health nurse was established by identification of at least one skin lesion consistent with primary or secondary treponemal infection [2].* ” We have addressed the question about sexual behaviour/activity under point 7 below as we believe it relates to participants’ data.

6. Line 180 – add ‘by’ before ‘Glatz’

To address one of the points raised by Reviewer#2, we inserted a “*Multiplex PCR*” subsection in the Methods section page 7, lines 151-154. This can now be read as follows: “*This multiplex PCR was performed at the University Hospital Zurich, Switzerland, with the Roche LightCycler 96 (Roche Diagnosis, Switzerland) targeting the 16S ribosomal RNA gene [28] for both T. pallidum and H. ducreyi, and glycoprotein B (gB) region of HSV-1 and -2 as described by Glatz and colleagues [28] with few alterations as outlined below.* ”

7. What additional data was collected from the participants? In the results data is presented on ethnicity. Please add in the method section.

We have modified the Sample and data collection section of the Methods, where we have added the following paragraph page 6, lines 122-126: “*Age and sex were recorded for each participant. Additionally, participants were asked to self-define their ethnicity. However, no information related to sexual behaviour or activity was recorded, since this is beyond the scope of the present research. Individual participants remained anonymous, i.e. the authors had no access to information that could identify individual participants during or after data collection.*” We believe this also addresses Reviewer#1 point 5 above about sexual behaviour and activity.

Results
1. The information presented in figures 3, 4 and 5 can be summarized in one table including statistical results. Also the demographics on age and sex as described in the first paragraph can be included.

We agree with Reviewer#1 here, and replaced Figures 3 and 5 by Tables 1 and 3 in the revised version of our manuscript. Table 1 summarises the data on the number of lesions with negative and positive point-of-care tests, the detection of *T. pallidum*, *H. ducreyi*, HSV-1 and HSV-2 with multiplex-PCR by study sites. Table 3 shows the detection of *T. pallidum*, *H. ducreyi*, HSV-1 and HSV-2 with multiplex-PCR in relation to whether lesions were negative or positive with the point-of-care test. We find the information displayed in both Tables is complementary and cannot be understood easily in a single table.

Figure 4 was showing the distribution of lesions that were negative and positive on the point-of-care tests according to recruited participant’s self-defined ethnicities. We agree this Figure was not displaying the message we wanted to convey appropriately and we have modified it so that this Figure 4 is now Fig 2 in our revised version. In this revised Fig 2, the distribution of recruited participant’s self-defined ethnicities according to study sites and metropolitan or municipal areas is shown. This is simply to highlight that even within a region like the Ashanti region in Ghana, there is not one single community affected by skin ulcers, but more than one. Addressing this may help designing eradication and elimination policies better. This also addresses one of Reviewer#2’s comments.

2. Line 217 – “Participants were asked to self-define their ethnicity.” Move this information to the method section.

As detailed when addressing Reviewer#1 point 7 above, this sentence now appears in the Methods under the updated Sample and data collection section page 6, lines 122-123and read as follows: “*Additionally, participants were asked to self-define their ethnicity.*”

3. After mentioning that the ethnicity data comes from self-defining, this does not need to be repeated throughout the manuscript.

This has been removed where it appeared multiple times, and it now appears in the Material and methods under the updated Sample and data collection section page 6, lines 122-123, but also in the caption of Fig 2, so that the Fig is clearly labeled for the reader.

4. If the confidence intervals are considered relevant, please add this information to the summarized table as suggested earlier or, if the authors disagree with the suggestion, add this to figure 3.

Reviewer#1 raises a good point here and we have changed our mind about showing the confidence intervals for the seroprevalence as the sample sizes per location were very heterogeneous (from 2 to 65) and we were not really using those confidence intervals further. We therefore show the numbers and associated proportions/percentages, but have removed the confidence intervals as we don’t think they were bringing any more meaningful information.

5. The paragraph on seroprevalence only show percentages, please add full numbers as well.

We appreciate this section, describing observed seroprevalence, was not clear, as some of the numbers were written in parentheses in the first paragraph, next to percentages, and further down only percentages were referred to. For sake of clarity, we have clarified this and wrote numbers first, then the corresponding percentage. These paragraphs of the Results now on pages 12 and 13, lines 235-253 read as follows: “*When combining participants with skin lesions consistent with either treponematose, the observed treponematose seroprevalence among study participants in the Ashanti region was 24/156 (15.4%). When yaws-like and syphilis-like lesions were considered separately, the observed seroprevalence among study participants was 19/110 (17.3%) and 5/46 (10.8%), respectively (Table 1). Among the study sites where yaws-like lesions were recorded, Mpobi had the highest seroprevalence, followed by Adweratia and Bebu, with seroprevalences of 7/23 (30.4%), 11/65 (16.9%) and 1/9 (11.1%), respectively (Table 1).*

*Interestingly, the pairwise comparison between the seroprevalence of Adweratia (11/65 ; 16.9%) and Mpobi (7/23 ; 30.4%), located in the same district (Afigya Kwabre South district), did not show any significant difference (Bonferroni adjusted p-value = 0.2). However, the seroprevalence of Bebu (1/9 ; 11.1%), located in the Atwima Kwanwoma district, and of Mpobi (7/23 ; 30.4 %) was significantly different (Bonferroni adjusted p-value = 0.008). Even though the seroprevalence of Adweratia (11/65 ; 16.9%) was higher than that of Bebu (1/9 ; 11.1%), the difference was not significant (Bonferroni adjusted p-value = 0.8). None of the participants recruited from the remaining three sites (Brofoyedu, Atia and Kotei) showed positive DPP tests. This suggests that yaws was more prevalent among study participants in the Afigya Kwabre South district (Adweratia and Mpobi) in the Ashanti region of Ghana compared to the other sites sampled. Even though the seroprevalence of syphilis at the SSH (4/27 ; 14.8%) was almost 3 times higher in comparison to that of KATH (1/19 ; 5.2%), this difference was not significant.*”

6. The pairwise analysis of seroprevalence per region may be easier to follow by using a heatmap-like table or figure in order for the readers to have an overview of what is being explained.

We have shortened this description and as suggested above (Results - Reviewer#1 point 1), this information has been summarised in Table 1 in the revised version of the manuscript. This Table presents the distribution of positive and negative point-of-care tests by study site, the corresponding seroprevalence and the results of the multiplex-PCR.

Discussion
1. The discussion section is too long (10 pages) and should be shortened to half its size.

We appreciate the discussion includes aspects that go beyond the results presented. Some of these aspects, including investigating the prevalence of yaws and syphilis together, have as far as we know not been integrated within a single study before. We have moved some aspects that were discussed in the original version submitted into the Supporting Information, including the information relating to original Figure 1, now S1 Fig, some of the explanations on recombination and congenital syphilis (S3 File). However, following both Reviewers’ comments we have had to expand on the limitations of the research and results presented here and, as a consequence, the Discussion does not appear any shorter. Please note that PLOS ONE does not request a minimum number of pages for their research articles.

2. The serological assay was positive in a low percentage of the total suspected lesions of yaws and syphilis. What was the cause of the lesions if not these pathogens?

As summarised page 12, “*when combining participants with skin lesions consistent with either treponematose, the observed treponematose seroprevalence in the Ashanti region was 24/156 (15.4%).*” Because only participants with clinical diagnosis consistent with treponemal lesions were recruited, it could have been expected that all these lesions (156/156) are indeed treponemal lesions and that the associated antibodies would be detected on the DPP test. There are however a few reasons why the suspected treponemal lesions may not be seropositive.

As mentioned pages 12 and 15, that those lesions that were serologically negative were indeed treponemal lesions cannot be entirely excluded since serological tests cannot detect early stages of treponemal infections. Additionally, it is known that pathogens other than *Treponema pallidum* species can cause skin lesions similar to those observed in cases of treponemal infections. These pathogens include *Haemophilus ducreyi*, herpes simplex virus -1 (HSV-1) and -2 (HSV-2), that were targeted with our multiplex-PCR approach. We anticipated that this approach would detect both early stages of treponemal infections and alternative pathogens causing skin lesions that resembles treponemal skin lesions, thereby explaining serologically negative tests. Out of the 156 tests performed, 132 were serologically negative and in only 23 of those was the presence of *T. pallidum*, *H. ducreyi*, HSV-1 and HSV-2 identified by amplification of their DNA with our multiplex-PCR approach. This leaves 109 serologically negative tests whose causative agent(s) remains to be identified.

We have explained in the Limitations section of the discussion pages 22 and 23 lines 436-458, the various reasons why the DNA extracted from the lesions sampled may not have been of the best quality by the time the multiplex-PCR was performed. It is therefore possible that the pathogens targeted by our multiplex-PCR approach, including *T. pallidum*, were responsible for some of these 109 negative tests. But more importantly, it is possible that additional pathogens, that were not targeted by our PCR approach, were responsible for those lesions that resemble treponemal lesions.

It appears misleading to envisage that lesions are caused by a single pathogen, and, at the time when sampling of lesions occurs, it seems unlikely to distinguish between the pathogen responsible for the initial lesion, i.e. opening of the skin, from the cohort or ecosystem of pathogens that may benefit from the lesion and consequently contribute to the development of this lesion. Performing 16S RNA gene sequencing would enable identifying the set of microorganisms co-infecting lesions, and investigating if those lesions where *T. pallidum* species occur show a significantly distinct sets of pathogens than those where *T. pallidum* doesn’t occur.

3. Lines 354 – 361 – The paragraph on congenital syphilis may be removed. The manuscript provides important data on prevalences, but its relevance to the elimination of congenital syphilis can be mentioned in one sentence.

We believe congenital syphilis is related to the prevalence of treponematoses, but we appreciate that our discussion was long. We have shortened this paragraph into two sentences (page 16, 319-322) with a reference to the Supporting Information file where we discuss various aspects of concerns for the eradication of yaws and elimination of syphilis (S3 File). The sentences on congenital syphilis reads as follows: “*The 10.8%* T. pallidum *seroprevalence among study participants with syphilis-like lesions confirms active or latent syphilis in these communities in Ghana. These infected participants, from a sexually active age range, could transmit the* T. pallidum *infection to an unborn baby, leading to congenital syphilis (S3 File).*”

4. I agree that the co-occurrence of syphilis and yaws in the same geographical area is interesting. Paragraph 362 – 379 should include information on Treponema pallidum subsp endemicum.

We thank Reviewer#1 for bringing this into our attention. This manuscript, and its associated research, focusses on the treponemes that have been reported in Ghana. *Treponema pallidum endemicum* has been reported in the Arabian Peninsula, more specifically in Iraq, Syria, and Saudi Arabia (Csonka and Pace, 1985). The African countries where *T. p. endemicum* has been reported include Mali, Niger, Burkina Faso and Senegal (Burke, 1985; Julvez, Michault and Kerdelhue, 1998). However, *T. p. endemicum* has not yet bee reported in Ghana.

However, the approaches we have used do not allow to distinguish between *T. pallidum* subspecies and we cannot, at this stage, exclude that the lesions associated to yaws could actually be bejel lesions (caused by *T. p. endemicum*). We have added the following sentences in the Limitations section of the Discussion page 22, lines 459-473 to address this: “*Another limitation worth mentioning relates to the pathogens targeted with multiplex-PCR here. The multiplex-PCR protocol applied has been developed for routine clinical practice targeting only four of the pathogens known to cause lesions similar to those observed in cases of yaws and syphilis, i.e.* T. pallidum*, HSV-1/ 2 and* H. ducreyi *[28], while other pathogens, including* M. ulcerans *and* C. trachomatis*, can also be found in such lesions in Ghana. Our study emphasises the need of designing sensitive and efficient multiplex-PCR protocols that could identify sets of pathogens found in specific types of lesions (for example pathogens causing skin ulcers).*

*In this context, this study focusses on the* T. pallidum *subspecies that have, to our knowledge, been reported in Ghana. However, lesions similar to yaws can also be observed in cases of bejel, another non-venereal treponematose caused by* T. p. endemicum*.* T. p. endemicum *has been reported in the Arabian Peninsula, more specifically in Iraq, Syria, and Saudi Arabia [54]. The African countries where* T. p. endemicum *has been reported include Mali, Niger, Burkina Faso and Senegal [55,56]. The approaches we have used here do not allow to distinguish between* T. pallidum *subspecies and we cannot, at this stage, exclude that the lesions associated to yaws could actually be bejel lesions.*”

5. Paragraph on recombination should be removed as this is outside of the scope of this manuscript.

Even though we appreciate our discussion is lengthy and the focus of our study is not recombination, we do not fully agree here with Reviewer#1. Much remains unknown about the biology and evolution of treponemes, and recombination is an essential biological process that generates diversity and contributes to species adaptation and spread of pathogens to various hosts. While recombination patterns between treponeme species and subspecies have been evidenced, as far as we know, that one individual could have been infected with *T. p. pertenue* as a child and by *T. p. pallidum* later in life has neither been eluted to before, nor been observed. This is why we believe it matters that our explanations about recombination are as explicit and clear as possible, with this example of the individual recruited for yaws-like lesions while potentially being a sexually active participant. We have however deleted most of the paragraph about recombination and replaced it with the single sentence: “*Variants resulting from recombination can pose diagnostic challenges because they can involve changes in conserved regions of DNA/RNA or proteins [13,20–24], which tend to be the molecular targets of both serological and molecular diagnose [24,35] (see S3 File for extensive discussion on the impact of recombination).*”

6. The limitation section could be more extensive including limitations in sample size per location, limited behavioral data, the use of a multiplex PCR with possibly less sensitive results?

We agree with Reviewer#1 that our sample size per location is limited, and we have included a sentence that says so in our Limitations section of the Discussion (page 22 lines 431-432: “*This is an exploratory study, where the sample size per study site is relatively low ranging from 2 to 65 participants recruited.*”). We have included a sentence on the lack of behavioural data, although the purpose of this study was not to characterise the distribution of syphilis among specific groups, especially since sexual preferences may be tabooed in some cultural groups and the related information we may have gotten would likely be biased. The purpose of this study was to investigate whether yaws and syphilis can be identified together within a single region and address whether other pathogens commonly found in similar skin lesions can also be identified there. This is what our study shows. Furthermore, the PCR protocol has been designed and optimized for clinical routine practices in screening laboratories. This protocol has been shown to be highly specific and sensitive (see Glatz et al. 2014), and we don’t think these aspects should be questioned here. We did question other aspects of the PCR approach, especially the quality of the DNA that is likely to have been greatly affected by shipment services. This again may seem anecdotical in settings where clinical samples stay in the same facilities (just going into different rooms or buildings), but this is not what had happened here. The samples were collected kilometres away from the laboratory where the DNA was extracted, and then they were shipped thousands of kilometres away where the multiplex PCR was performed. The fundings available for this exploratory study were limited and decisions had to be made to trade-off between sampling schemes and molecular biology techniques applied. We believe, despite these limitations, our study emphasises that, in an area when it is accepted that microbiomes affect health, scientists cannot be thinking of skin lesions as being caused by a single pathogen at a time, but should rather consider the community of related microbes that could interact to prevent healing.

7. Looking at the seroprevalence, misdiagnoses of syphilis and yaws seem to be very common as other ulcer-causing pathogen were detected instead of T. pallidum. Only 17.2% of the yaws-like lesions and 10.8% of the syphilis-like lesions were positive by serological testing (and one yaws-like lesions by PCR). This would suggest that 82.8% and 89.2% are not yaws or syphilis and may thereby have been misdiagnosed. If that is correct then please rephrase 522 – 524 “The prevalence … lesions determined.” Change “co-occurrence” into “occurrence”.

We appreciate Reviewer#1’s comment, we had not considered this that way. We have rephrased these lines and the relevant sentences of the conclusion now read as follows on lines 479-486: “*The prevalence of the treponematoses was assessed among 156 individuals showing lesions consistent with the diagnoses of syphilis and yaws. A seroprevalence of 17.3% (19/110) and 10.8% (5/46) of* T. pallidum *in individuals with yaws-like and syphilis-like lesions, respectively, indicate the occurence of treponemal strains causing yaws and syphilis within the Ashanti region of Ghana. Other pathogens known to cause similar skin lesions were also identified.* H. ducreyi *was more prevalent in yaws-like lesions, while HSV-2 was more prevalent in syphilis-like lesions. This is the first study reporting the presence of HSV-1 in yaws-like lesions (2/110).*”

Reviewer #2: The manuscript “Prevalence of yaws and syphilis in the Ashanti region of Ghana and occurrence of H.ducreyi, Herpes simplex virus 1 and Herpes simplex virus 2 in skin lesions associated with treponematoses” by Yaw Agyekum Boaitey and colleagues aims to show the importance of correct diagnosis of the causative agent of ulcer lesions. The results of this study show presence of multiple pathogens present in ulcers of patients in Ghana, causing similar symptoms, and the need for applying the right treatment to prevent overuse of antibiotics. However, this reviewer has several concerns.

1. Figure 1: The description is very long and would be better suited for Methods section, as it describes the process in which it has been built. In boxplot part of the figure, it also shows results of the study, but the figure is placed in the Introduction section. It is not needed to show the data on endemic countries in a figure form. I would suggest either removing the figure, moving it to the Supplementary materials or keeping only the boxplot part of the figure and moving it to the Results section. Also, add a reference to the Supplementary table into the text.

This is similar to one of Reviewer#1’s suggestions. We appreciate this comment and, in our revised version, we have moved this Figure to the Supporting Information. Moving this Figure and the information associated to it also contributes to making the overall manuscript more concise (one of Reviewer #1 General comments). This Figure is now S1 Fig and the corresponding description can be found in S3 File.

2. Lines 124 to 127: Why did authors select these three pathogens to be analysed in their study? On lines 120 to 123, they mention additional pathogens able to cause skin lesions similar to those of treponemes. What was the reason behind selection of the pathogens? This needs to be addressed.

We appreciate the reason why we selected these pathogens was not clearly explained in our first version of the submitted manuscript. While all the pathogens mentioned in the introduction occur in Ghana (i.e. *Mycobacterium ulcerans*, *Chlamydia trachomatis*, *Treponema pallidum*, herpes simplex virus types 1 or 2 (HSV-1/ 2) and *Haemophilus ducreyi*), a multiplex-PCR protocol has been developed for routine clinical practice targeting only four of those, i.e. *T. pallidum*, HSV-1/ 2 and *H. ducreyi* (Glatz et al. 2014, reference [28] in the manuscript reference list). We have rephrased this sentence of the introduction (page 4, lines 75-78) as follows: “*In this study, we explored the prevalence of syphilis and yaws in the same geographical area, as well as the presence of three other pathogens known worldwide to be causing lesions similar to those of yaws and syphilis and known to occur in Ghana, i.e. HSV-1, HSV-2,* H. ducreyi*, and for which a multiplex PCR protocol exists [28].* ” We have also inserted the following sentence page 7, lines 149-151 in the “*Multiplex PCR*” subsection of the Methods “*A multiplex-PCR protocol previously developed for routine clinical practices specifically targeting molecular identification of* T. pallidum*, HSV-1/ 2 and* H. ducreyi *was applied [28].*”

We believe Reviewer#2 raises an important aspect here as multiplex-PCR targeting various organisms are challenging to set up, and developing an efficient and sensitive multiplex PCR including all those pathogens was beyond the scope of the current research. We however include this in the updated version of the Limitations section of our revised manuscript version (page 23, lines 459-465) as follow: “*Another limitation worth mentioning relates to the pathogens targeted with multiplex-PCR here. The multiplex-PCR protocol applied has been developed for routine clinical practice targeting only four of the pathogens known to cause lesions similar to those observed in cases of yaws and syphilis, i.e.* T. pallidum*, HSV-1/ 2 and* H. ducreyi *[28], while other pathogens, including* M. ulcerans *and* C. trachomatis*, can also be found in such lesions in Ghana. Our study emphasises the need of designing sensitive and efficient multiplex-PCR protocols that could identify sets of pathogens found in specific types of lesions (for example pathogens causing skin ulcers).*”

3. In Methods, Sample and data collection: The authors need to describe the process of acquiring samples and diagnosis determination in a greater detail.

We have added this information in the section referring to Sample and data collection of the Methods (pages 5 and 6 lines 110-126). The relevant paragraphs now read as follow: “*Since yaws affects mainly children (≤15 years of age with reported peak incidence occurring in children aged 6-10 years) living in deprived rural areas, we enrolled children from six deprived communities in the Ashanti region. Participants from 5-17 years of age gave their verbal assent before their guardians were asked to sign the informed consent form. Participants who were 5 to 17 years of age with a minimum of one clinical lesion consistent with primary or secondary treponemal (yaws) infection [2] were enrolled for yaws-like lesion samples.*

*Individuals commonly affected with syphilis are young adults and adults and, in Ghana, they usually report to hospitals or pharmacies. This is why two government hospitals were visited for recruiting adult participants with syphilis-like lesions. Participants who were 18 years and above with a minimum of one clinical lesion consistent with primary or secondary treponemal infection (syphilis) were enrolled for syphilis-like lesion samples.*

*Age and sex were recorded for each participant. Additionally, participants were asked to self-define their ethnicity. However, no information related to sexual behaviour or activity was recorded, since this is beyond the scope of the present research. Individual participants remained anonymous, i.e. the authors have no access to information that could identify individual participants during or after data collection.*”

Was there an age criterium when selecting the patients for each group (see lines 161 to 165)? Were there patients with suspected yaws and syphilis in both rural and urban locations, and were they included? If not, why?

As explained in the revised version of the manuscript copied above, we did not look for syphilis-like lesions in rural communities. Experience from health authorities we consulted before the start of the project explained that since syphilis usually involves sex, most people affected feel uncomfortable talking about it and they either visit the hospital or the pharmacy for treatment. We would have been unlikely to recruit participants with syphilis-like lesions in rural communities, where clinics and hospital are not readily available. This informed our decision to recruit participants with syphilis-like lesions from hospitals.

What were the major factors deciding the clinical diagnosis and separation into yaws-like or syphilis-like lesion groups? Especially in the samples included into syphilis-like lesion group, from patients that had lesions on arms and belly, which is location more typical for yaws lesions.

As mentioned in the text copied from our revised version, this was based on the finding of a minimum of one clinical lesion consistent with primary or secondary treponemal infection (yaws in children and syphilis in adults). “Lesions” could be ulcers (primary stage) or rash / maculopapular exanthema (secondary stage), which in case of syphilis are often found on belly and arms. Lesions on arms and belly are not classical for yaws. The distribution of skin ulcers on body parts for yaws-like and syphlis-like lesions is shown on Table 2.

4. Why were samples divided into these two groups, if there are many other pathogens that cause lesions similar to ones caused by treponematoses, a point which had been raised by the authors in the Introduction?

Our samples were divided into yaws-like and syphilis-like lesions because these treponematoses, both occurring in Ghana, are at the core of this research, while the other common pathogens that cause similar lesions are not. In this research, we want to emphasise that, while syphilis and yaws affect different cohorts, since they are caused by bacteria that are 99.8% genetically similar (Cejkova et al., 2012), both yaws and syphilis should be diagnosed and treated in regions where yaws is endemic for achieving yaws eradication and syphilis elimination. In addition, it is known that other pathogens cause skin lesions similar to those attributed to treponemes. In fact, the multiplex-PCR protocol (Glatz et al. 2014) we apply has been developed on specimen from syphilis-like lesions, i.e. genital lesions, as the first sentence from Glatz et al. (2014) states: “*Treponema pallidum*, herpes simplex virus types 1 or 2 (HSV-1/ 2) and *Haemophilus ducreyi* are, worldwide, the most common sexually transmitted pathogens that cause ulcers of the genital, anal and oropharyngeal region [1,2].” However, it is the first time this TP-HD-HSV1/2 PCR is applied on yaws-like lesions, even though *H. ducreyi* has also been reported in yaws-like lesions. This is another argument in favour of diagnosing and treating both yaws and syphilis in countries where yaws is endemic.

5. Was the seropositivity based on the density of the treponemal line in DPP test or just by visual presence of the line? Also, please include the sentence from lines 337 to 338 into methods, to be clear about what samples were considered serologically positive.

Seropositivity was based on the presence of a line, whether faint or dense, since, as explained by the manufacturer (Chembio Diagnostic Systems Inc. Medford, New York), faint or deep line on the test line should be considered positive as long as the control line appears. We followed the manufacturer’s instructions here.

We have edited the following section of the Methods, “*Clinical diagnosis and seroprevalence*” subsection, to be clear about what samples were considered serologically positive (page 7): “*Seropositivity for either treponematose was determined when both treponemal and non-treponemal test lines were observed. This applies to both yaws-like and syphilis-like participants. In other words, participants we refer to as “DPP-positive” in this study showed both the treponemal and the non-treponemal test lines on their point-of-care test.*”

6. Line 178 to 180: A brief description of the multiplex PCR is needed. What were the targets for each pathogen and conditions of the reaction?

A “*Multiplex PCR*” subsection (page 7, lines 149-160 ) has been added in the Methods section, and a description of the protocol has been inserted as follows: “*A multiplex-PCR protocol previously developed for routine clinical practices specifically targeting molecular identification of* T. pallidum*, HSV-1/ 2 and* H. ducreyi *was applied [28]. This multiplex PCR was performed at the University Hospital Zurich, Switzerland, with the Roche LightCycler 96 (Roche Diagnosis, Switzerland) targeting the 16S ribosomal RNA gene [28] for both T. pallidum and H. ducreyi, and glycoprotein B (gB) region of HSV-1 and -2 as described by Glatz and colleagues [28] with few alterations as outlined below. Briefly, 20 µL reactions consisted of 10 µL Takyon No Rox Probe MasterMix (Eurogentec, Belgium), 0.5 µM of each primer, 0.1 µM of each probe, 1 µL internal control and 5 µL of extracted DNA. Reaction conditions were activation of uracil-N- glycosylase at 50 °C for 2 minutes, pre-denaturation at 95 °C for 3 minutes, 45 cycles of denaturation at 95 °C for 10 seconds and annealing and amplification at 63 °C for 45 seconds. Cycle threshold (Ct) value less than 40 cycles was taken as positive.*”

Also, since the authors claim that one aim of the study was to assess the prevalence of treponematoses and since there is a possibility of misdiagnosis when using only serology, why authors did not use a target for the PCR that could differentiate between the two treponemal subspecies and confirm less reliable clinical diagnosis? Serology and non-specific PCR will also not allow for detection of possible coinfection by both yaws and syphilis, which is speculated in the Discussion.

One of the objectives of this study was to screen with the multiplex PCR for *T. pallidum* and other organisms that cause similar lesions in a first step and then use multi-locus sequence typing (MLST) on *T. pallidum* amplified DNA to differentiate between the two species, i.e. yaws and syphilis treponemes, in a second step. However, after the multiplex PCR, we had only one sample with *T. pallidum* DNA being amplified, and the concentration of this DNA was low. This is why we did not perform the MLST.

7. Why did the authors not use a control in the multiplex PCR, for example detection of the patients' DNA, to differentiate between negative result and low quality sample?

We agree with the reviewer that using a human DNA control would have been a good choice. Instead, we used an inhibition control as internal control as this is done routinely in the lab of the University Hospital Zurich where the PCRs were performed. We added this to the Limitations (page 21, lines 443-451): “*In order to assess whether other pathogens commonly found in skin lesions similar to those produced by* T. pallidum *subspecies could also be present in those skin lesions in Ghana, we applied a multiplex PCR protocol with high specificity and sensitivity established for the detection of worldwide transmitted pathogens in routine clinical practice [28]. However, DNA conservation in samples collected in so-called routine clinical practices cannot directly compare to DNA conservation of the samples collected in the context of this study. One possibility for distinguishing between negative result and low-quality sample would have been to add a control targeting human DNA in the multiplex PCR, as well as the inhibition control already integrated in this protocol.*”

8. Figure 4, showing ethnicity of the study participants is not needed, or could perhaps be combined with Figure 2 or 3, to show the distribution of the ethnicities in relation to rural/urban locations.

Figure 4 was showing the distribution of lesions that were negative and positive on the point-of-care tests according to recruited participant’s self-defined ethnicities. We agree this Figure was not displaying the message we wanted to convey appropriately, and we have modified it so that this Figure 4 is now Fig 2 in our revised version. This also addresses one of Reviewer#1’s comments. In this revised Fig 2, the distribution of recruited participant’s self-defined ethnicities according to study sites and metropolitan or municipal areas is shown. This is simply to highlight that even within a region like the Ashanti region in Ghana, there is not one single community affected by skin ulcers, but more than one. Addressing this may help designing eradication and elimination policies better.

9. Lines 437 to 442: Why do authors state that all the syphilis-like lesion samples with DNA amplification by multiplex PCR were positive for HSV-2, if the prevalence of HSV-2 in these samples was 28.3% (13/46)? Please clarify what is meant by this sentence.

This has been clarified and worded as follows (page 18, line 378): “*Amongst the syphilis-like lesion samples, 13 (28.3%) were positive for HSV-2 (Tables 1 and 3).*”

10. Since authors call for the molecular approaches to be implemented in yaws-endemic countries to diagnose the cause of the lesions, could they discuss what would be the appropriate pathogens to test for?

As was mentioned above (Reviewer#2 point 2) as well as in the Introduction of the manuscript, other pathogens causing similar skin lesions to those caused by treponemes are found in Ghana, including those not targeted by the multiplex PCR protocol applied here, i.e. *M. ulcerans* and *C. trachomatis*. It is therefore possible that those skin lesions that were not serologically positive with the DPP test and that did not show DNA amplification of any of the targeted pathogens would have been caused by other pathogen(s). This has been made more explicit in the Limitations of the revised version of the manuscript (page 23, lines 460-465).

In this study, the authors were detecting treponemes, as well as H. ducreyi and HSV-1 and HSV-2. Is there other pathogens present in Ghana, that could cause the lesions, but were not tested for, or do authors think that the negativity of the multiplex PCR for the analysed pathogens was mostly caused by the bad sample quality, that could have resulted from factors they mention in Limitations?

As stated above in answer to one of Reviewer#2’s previous comments, Reviewer#2 raises an important aspect here as multiplex-PCR targeting various organisms are challenging to set up, and developing an efficient and sensitive multiplex PCR including all those pathogens was beyond the scope of the current research. We however include this in the updated version of the Limitations section of our revised manuscript version as follow: “*Furthermore, the multiplex-PCR protocol applied has been developed for routine clinical practice targeting only four of the pathogens known to cause lesions similar to those observed in cases of yaws and syphilis, i.e. T. pallidum, HSV-1/ 2 and H. ducreyi (Glatz et al. 2014), while other pathogens, including M. ulcerans and C. trachomatis, can also be found in such lesions in Ghana. Our study emphasises the need of designing sensitive and efficient multiplex-PCR protocols that could identify sets of pathogens found in specific types of lesions (for example pathogens causing skin ulcers).*”


Minor comments:
1. Line 48: Yaws does affect not only children between 2 and 15 years of age, please rephrase.

This has been rephrased on line 48 as follows: “*Yaws is a neglected tropical disease which mostly affects the skin of children ≤15 years of age [1].*”

2. Please unify the style in which the treponemal subspecies are referred to throughout the text. Authors use Treponema pallidum subsp. Pertenue, T. pallidum subsp pertenue, T. p. pertenue and TPE (without previous reference) at different places (same for subspecies pallidum).

This has been corrected and homogenised throughout the manuscript.

3. Please label the A, B and C parts of Figure 2 accordingly, as described in text.

This has been done. Figure 2 is now Fig 1 in the revised version of our manuscript, and labels A, B and C have been inserted.

4. Add reference to the Supplementary Data to the Results and describe the data included there.

At the end of the manuscript, a section called Supporting Information has been added and the following files appear with the corresponding descriptions:

*“Supporting Information*

*S1 Fig. Prevalence of yaws and syphilis estimated from studies published between 1991 and 2020. (A) Prevalence of yaws and syphilis estimated from 89 peer-reviewed studies (number of positive cases for yaws or syphilis divided by the total number of participants per study). Out of the 15 countries known to be endemic for yaws, 13 are represented in the selected peer-reviewed articles reporting the prevalence of yaws or syphilis (no data published between 1991 and 2020 on the prevalence of yaws and syphilis from Cameroon and Liberia that fitted our criteria). Only four countries showed estimates of the prevalence of yaws and syphilis, from independent studies, i.e. Ecuador, Ghana, Indonesia, Nigeria. For comparison, blue triangles show the seroprevalence of yaws and syphilis estimated in the Ashanti region of Ghana from this study. There is no significant difference between any of the estimated prevalence of yaws and syphilis (Wilcoxon Rank Sum test p-values are 0.77 between the published prevalence of yaws and syphilis (globally); 0.67 between the published prevalence of yaws and syphilis in Ecuador; 0.70 between the published prevalence of yaws and syphilis in Ghana; 1 between the published prevalence of yaws and syphilis in Indonesia; 0.50 between the published prevalence of yaws and syphilis in Nigeria. The Chi-squared test p-value is 0.44 between the observed seroprevalence of yaws and syphilis in the Ashanti region). (B) Median of the estimated prevalence of yaws by country in studies published between 1991 and 2020. The borders of the four countries where the prevalence of both yaws and syphilis have been reported (Ecuador, Ghana, Indonesia, Nigeria) are represented in red. (C) Median of the estimated prevalence of syphilis by country in studies published between 1991 and 2020. The borders of the four countries where the prevalence of both yaws and syphilis have been reported (Ecuador, Ghana, Indonesia, Nigeria) are represented in red.*

*S1 Table. Data collected from the 156 participants from the Ashanti region of Ghana, this study.*

*S2 Table. Data from 89 peer-reviewed articles reporting the prevalence of yaws and syphilis.*

*S1 File. Inclusivity in global research questionnaire.*

*S2 File. Rscript for reproducing data analyses and graphs, including maps.*

*S3 File. Extensive discussion with references.*

*S4 File. References for the 89 peer-reviewed research articles in S2 Table.“*

We hope we have addressed the Editor’s and both Reviewers’ comments appropriately and that our manuscript now meets PLOS ONE publishing criteria.

Best regards,

Pascale Gerbault, on behalf of the co-authors.
