# Supplementary material for: Prevalence of yaws and syphilis in the Ashanti region of Ghana and occurrence of H. ducreyi, herpes simplex virus 1 and herpes simplex virus 2 in skin lesions associated with treponematoses
Source: PLoS One. 2024 May 22;19(5):e0295088. doi: 10.1371/journal.pone.0295088 (PMC11111032; doi:10.1371/journal.pone.0295088)
Supplement: Attachment — Submitted filename: Boaitey_et_al_ResponseToReviewers2024.05.03.docx [file pone.0295088.s010.docx]

PONE-D-23-37271R1 - REBUTTAL LETTER -

Prevalence of yaws and syphilis in the Ashanti region of Ghana and occurrence of H. ducreyi, herpes simplex virus 1 and herpes simplex virus 2 in skin lesions associated with treponematoses.

PLOS ONE

Dear PLOS ONE Academic Editor Dr Sylvia Maria Bruisten,

We thank Reviewer#2 who raised additional points during the review process and you for your comments to further improve our manuscript PONE-D-23-37271R1 for publication in PLOS ONE. This rebuttal letter addresses these points, left in black font below. Our responses to each of those points is in blue font. When we cite sections of our revised manuscript, the text appears in black and italic font. When we cite line numbers, we refer to the revised versions (of the manuscript or the S3 File supplementary information) without Track changes.

Journal Requirements:

Please review your reference list to ensure that it is complete and correct. If you have cited papers that have been retracted, please include the rationale for doing so in the manuscript text, or remove these references and replace them with relevant current references. Any changes to the reference list should be mentioned in the rebuttal letter that accompanies your revised manuscript. If you need to cite a retracted article, indicate the article’s retracted status in the References list and also include a citation and full reference for the retraction notice.

We realised that reference 31 [Rod James. Guidelines on microbiological wound swabbing. 2018;(September)] that was cited for sampling the lesions with Dacron swabs was not accessible any longer. We cite here instead Munson et al. (2019). We have replaced our previous reference 31 with this one: [Munson M, Creswell B, Kondobala K, Ganiwu B, Lomotey RD, Oppong P, Agyeman FO, Kotye N, Diwura M, Ako EP, Simpson SV. Optimising the use of molecular tools for the diagnosis of yaws. Transactions of The Royal Society of Tropical Medicine and Hygiene. 2019 Dec 1;113(12):776-80.]. This article shows amplification of DNA from yaws-like lesions after sampling of skin ulcers with dry Dacron swabs, as used in our study.

6. Review Comments to the Author

Please use the space provided to explain your answers to the questions above. You may also include additional comments for the author, including concerns about dual publication, research ethics, or publication ethics. (Please upload your review as an attachment if it exceeds 20,000 characters)

Reviewer #1: The author addressed all comments providing arguments to adjust or compromise on the suggestions that were made.

We thank Reviewer #1 for their understanding and appreciation of our amendments to the original version of the manuscript.

Reviewer #2: The authors addressed majority of the concerns raised by this reviewer. However, this reviewer has several minor points.

We thank Reviewer #2 for their adding further aspects of our manuscript that need clarifying. We believe we have addressed those points below.

Minor points:

In Fig. 2: Black text on a dark blue background is hard to read.
This black font colour has now been changed for grey. We have also checked this Figure with PACE tool as well and it reads fine. The S2 File showing the Rscript for reproducing data analyses and graphs, including maps, has also been updated accordingly.

In the Limitation section of this study, the authors should clearly state that the evidence for yaws and syphilis is solely based in clinical and epidemiological data and not on the molecular analysis of DNA.

Following Reviewer #2’s suggestion, we have inserted three sentences addressing this in the Limitation section lines 443-448 of the revised manuscript and read as follows:

“*It should be stressed that the identification of yaws-like and syphilis-like lesions was based solely on clinical and epidemiological observation. The names of the lesions however are not used in our assessment of the prevalence of either treponematose and we do not think this affects our results. The molecular analysis of DNA was then performed as to provide a complementary examination of these lesions.*”

Indeed, we are not using the names of the diseases as diagnosis evidence for those diseases, i.e. we are not using clinical and epidemiological data to assess the prevalence of either treponematose. We are using the names of the diseases in association to what the skin lesions looked like, i.e. “yaws-like lesions” and “syphilis-like lesions”. This is because when a lesion is examined, based on clinical and epidemiological observation, lesions that have been caused by treponemes may actually look like lesions caused by other pathogens, including *H. ducreyii* or herpes simplex viruses 1 or 2. This is why we used a point-of-care test in order to identify the presence of treponeme antigens in those lesions. We further want to emphasise that molecular analysis of DNA from lesions can miss the diagnosis of a treponematose (later stage of disease or very early stage of disease when treponeme DNA quantity is low) - and equally serological diagnosis can miss diagnosing a treponematose (early stage of the disease). We therefore do not think that a diagnosis based on clinical and epidemiological observation of lesions alone or a diagnosis based on molecular analysis of lesions alone is better than the other. We rather want to use them both as complementary tools that inform on the possible pathogens present in skin lesions that look like yaws and syphilis lesions.

The section of supplementary discussion „T. pallidum infections and congenital syphilis“ is not directly related to the study presented here and does not bring any new information. Moreover, the section of supplementary discussion focusing on recombination is perhaps too long, since in this study the co-infection is just a speculation, as the authors presents no evidence of it. It would be enough to mention the possibility of recombination including recombination in the primer site(s).

We agree with Reviewer #2 that the paragraph on “*T.pallidum* infections and congenital syphilis” did not bring much to the debate as it read. However, even though we do not assess congenital syphilis in participants, we do not agree that congenital syphilis is not related to the study we present here. We have consequently reworded the section on “*T. pallidum* infections and congenital syphilis” in the supplementary information to clarify why we think it matters to consider syphilis, congenital syphilis and yaws together. We have added one more reference to support this opinion, reference number 7 in the supplementary information S3 File (Tsuboi M, Evans J, Davies EP, Rowley J, Korenromp EL, Clayton T, et al. Prevalence of syphilis among men who have sex with men: a global systematic review and meta-analysis from 2000-20. Lancet Glob Health. 2021 Aug;9(8):e1110-e1118). The corresponding paragraph lines 95-106 (S3 File) now reads as follows:

“*The 10.8%* T. pallidum *seroprevalence among study participants with syphilis-like lesions confirms active or latent syphilis in Ghana (Table 1). In this study, the median age of participants with syphilis-like lesions was 29 with minimum age of 19 and maximum age of 75, a sexually active age range. These infected participants could transmit their* T. pallidum *infection to an unborn baby, leading to congenital syphilis. WHO considers congenital syphilis as a public health problem [6]. To eliminate congenital syphilis, treating pregnant women is not sufficient: syphilis must also be eliminated from the general population. In fact, reducing the global syphilis incidence by 90% between 2018 and 2030 is one of the four ambitious targets detailed in the Global Health Sector strategy addressing sexually transmitted diseases [7]. We argue supporting accurate diagnosis of syphilis and specific assessment of the presence of its causative treponeme,* T. pallidum pallidum*, and of related treponemes, such as* T. pallidum pertenue *causing yaws, in countries such as Ghana, will be essential to achieving this Global Health Sector strategy target.”*

We have shortened the section on recombination in the supplementary information S3 File lines 108-133 deleting some of the details on the recombined strains, and making our speculative statement explicit. This section and this hypothesis - even speculative - are however important to develop here as it has never been explicitly eluded to before and it needs to be raised to be looked into further in future studies on treponematoses.

7. PLOS authors have the option to publish the peer review history of their article (what does this mean?). If published, this will include your full peer review and any attached files.

If you choose “no”, your identity will remain anonymous but your review may still be made public.

Do you want your identity to be public for this peer review? For information about this choice, including consent withdrawal, please see our Privacy Policy.

Reviewer #1: No

Reviewer #2: No

[NOTE: If reviewer comments were submitted as an attachment file, they will be attached to this email and accessible via the submission site. Please log into your account, locate the manuscript record, and check for the action link "View Attachments". If this link does not appear, there are no attachment files.]

While revising your submission, please upload your figure files to the Preflight Analysis and Conversion Engine (PACE) digital diagnostic tool, https://pacev2.apexcovantage.com/. PACE helps ensure that figures meet PLOS requirements. To use PACE, you must first register as a user. Registration is free. Then, login and navigate to the UPLOAD tab, where you will find detailed instructions on how to use the tool. If you encounter any issues or have any questions when using PACE, please email PLOS at figures@plos.org. Please note that Supporting Information files do not need this step.

We registered as PACE users, uploaded our Fig 1 and Fig 2 and checked they could be easily read and appeared clearly. We have then downloaded Fig 1 and Fig 2 back and uploaded these versions onto PLOS ONE Editorial Manager.

On the Track changes version of our revised manuscript, we have also made a few minor changes that are listed below:

- deletion of extra spaces lines 92, 102, 216, 265;
- clarification of what “seropositivity” refers to in this study by adding “antibody” and in the point-of-care test” line 137;
- adding “the latter” to clarify that the “the Upper East, Upper West and Northern regions” refer to “the Northerners” line 204;
- deletion of unnecessary “in” line 214 (Fig 2 caption);
- inserting “respectively” line 281 in the caption of Table 3 to clarify “DPP.negative” and “DPP.positive” definitions;
- updating the reference to Tables 1 and 3 line 314 instead of Fig 4 from an earlier version of the manuscript;
- ensuring the prevalence of yaws and syphilis estimated from multiplex-PCR in the text matches the reported percentage from Table 3 (0.6% and not 0.67%);
- there are various reasons why yaws and syphilis have been reported independently, we only cite one of them here, consequently we specify “one of the reasons why” line 332;
- we have inserted a comma line 334 (after co-occur);
- we prefer “different” instead of “distinct” line 347 in relation to treponeme subspecies;
- an “s” was missing at the end of “diagnose” line 350;
- the list of references has been updated line 372;
- a “-“ has been added lines 391 and 408 to make it clear the “2” refers to HSV-2;
- we prefer the use of “in addition” in the context of the text line 412 rather than “on the other side”;
- we think “notably” is more accurate than “noteworthy” line 417;
- line 424 we have replaced “and let” by “leading”;
- we realised we refer to DNA “preservation” and not “conservation” lines 460, 461, 465 and 468;
- line 477 we have replaced “of designing” by “to design”.

We hope we have addressed the Editor’s and Reviewer #2’s comments appropriately and that our manuscript now meets PLOS ONE publishing criteria.

Best regards,

Pascale Gerbault, on behalf of the co-authors.
